# Ciclopirox inhibits Hepatitis B Virus secretion by blocking capsid assembly

**DOI:** 10.1038/s41467-019-10200-5

**Published:** 2019-05-16

**Authors:** Jung-Ah Kang, Songwon Kim, Minji Park, Hyun-Jin Park, Jeong-Hyun Kim, Sanghyeok Park, Jeong-Ryul Hwang, Yong-Chul Kim, Yoon Jun Kim, Yuri Cho, Mi Sun Jin, Sung-Gyoo Park

**Affiliations:** 1 School of Life Sciences, Gwangju Institute of Science and Technology, Gwangju, 61005 Republic of Korea; 2 Department of Internal Medicine, CHA Gangnam Medical Center, CHA University School of Medicine, Seoul, 06125 Republic of Korea; 3 Department of Internal Medicine and Liver Research Institute, Seoul National University College of Medicine, Seoul, 03080 Republic of Korea

**Keywords:** Drug discovery, Antiviral agents, Hepatitis B virus, X-ray crystallography

## Abstract

Chronic hepatitis B virus (HBV) infection can cause cirrhosis and hepatocellular carcinoma and is therefore a serious public health problem. Infected patients are currently treated with nucleoside/nucleotide analogs and interferon α, but this approach is not curative. Here, we screen 978 FDA-approved compounds for their ability to inhibit HBV replication in HBV-expressing HepG2.2.15 cells. We find that ciclopirox, a synthetic antifungal agent, strongly inhibits HBV replication in cells and in mice by blocking HBV capsid assembly. The crystal structure of the HBV core protein and ciclopirox complex reveals a unique binding mode at dimer-dimer interfaces. Ciclopirox synergizes with nucleoside/nucleotide analogs to prevent HBV replication in cells and in a humanized liver mouse model. Therefore, orally-administered ciclopirox may provide a novel opportunity to combat chronic HBV infection by blocking HBV capsid assembly.

## Abstract (web summary)

Current treatments for chronic hepatitis B virus (HBV) infection are not curative. Here, the authors show that an antifungal drug, ciclopirox, inhibits HBV capsid assembly and synergizes with nucleoside/nucleotide analogs to prevent HBV replication in cells and in a humanized liver mouse model.

## Introduction

Hepatitis B virus (HBV) is a double-stranded DNA virus and a member of the Hepadnaviridae family of viruses^1,2^. Chronic HBV infection is a major global cause of hepatocellular carcinoma (HCC); chronic carriers have a >100-fold increased relative risk of developing HCC. The virion (called Dane particle) is a spherical particle 42 nm in diameter. It is composed of an icosahedral nucleocapsid and an envelope that consists of three HBV surface proteins and lipids from the host cells^3^. The HBV surface proteins are referred to as the large, middle, and small S proteins. The large S protein contains the preS1 region, which participates in the entry of the virus into host cells^2^. The preS1 region binds to the host protein sodium taurocholate cotransporting polypeptide (NTCP); thus when cells of hepatocyte-derived cell line,s such as HepG2 and Huh-7, were transfected with NTCP, they became susceptible to HBV^2^. The nucleocapsid consists of the HBV core protein complexed with the virus-encoded polymerase and the viral DNA genome. During HBV infection, the HBV envelope first fuses with the host plasma membrane, after which the nucleocapsid is released into the host cytosol and is delivered to the nuclear pore^4^. After passing through the nuclear pore into the nucleus, the viral genome is converted into a covalently closed circular DNA (cccDNA) molecule. This cccDNA encodes four overlapping open reading frames that produce seven proteins, namely, the large, middle, and small S proteins, the HBV core protein, the polymerase, and hepatitis B x (HBx) protein. The HBx protein has been implicated in hepatocarcinogenesis^5,6^.

HBV can be treated with nucleos(t)ide analogs (NAs) that inhibit the HBV polymerase, such as tenofovir (TDF) and entecavir (ETV); both are potent and have a high genetic barrier to drug resistance. However, they are unable to cure HBV because of the remarkable stability of the viral cccDNA intermediate, which is not directly targeted by NAs^7^. Treatment efficacy may be improved by combining NAs with peginterferon α2a (pegIFNα2a) in add-on or switch regimens. These combination therapies may act synergistically because they elicit both antiviral and immunomodulatory activities. Indeed, the TDF and pegIFNα2a combination induces hepatitis B surface antigen (HBsAg**)** loss better than either therapy alone. However, the combined rate is still only 9.1%^8^. In addition, many patients cannot tolerate pegIFNα2a^9^.

Thus, new therapies are needed for chronic HBV-infected patients. In particular, therapies that directly eradicate intrahepatic cccDNA and thereby clear persistent HBV infections. This goal has been greatly advanced recently by significant developments in understanding the HBV life cycle, including how the virus enters the host cell^10^, how cccDNA is formed^11^, and how the HBV capsid is assembled^12^. Insight into these mechanisms has led to the development of many novel agents against HBV that are being investigated. They include entry inhibitors^13^, such as Myrcludex-B, HBV capsid assembly inhibitors^14–16^, such as NVR 3–778, AT-61, AT130, and Bay 41–4109, and cccDNA inhibitors^17^, such as CCC-0975 and CCC-0346. Agents that boost the immune responses of the host^18–21^, such as GS-9620, SB 9200, GS-4774, and ABX203, are also being tested.

Early electron microscopy (EM) and crystallographic studies showed that the HBV core protein assembles to form two differently sized HBV capsids that consist of 180 and 240 HBV core proteins and have *T* = 3 (~5%) and *T* = 4 (~95%) icosahedral symmetry, respectively^22–25^. The core protein forms a dimer as the building block in assembling HBV capsid. It contains an N-terminal assembly domain (residues 1–149) and a C-terminal viral RNA-binding domain (residues 150–183). In the past decade, several technological advances have led to the identification of antiviral compounds that modulate assembly of the HBV capsid by the HBV core protein. These advances include the development of a simple purification method, generation of an assembly-competent construct (Cp149) containing only the N-terminal 149 residues, and the discovery that the Y132A mutant of Cp149 (Cp149-Y132A) does not assemble into capsids; instead, it forms thermostable dimers in solution and in the crystalline state, and yields crystals that diffract much more strongly than those of the wild-type core protein^12,26–30^. There is good evidence that Cp149-Y132A is a structural and functional homologue of the wild-type core protein. For example, while Cp149-Y132A itself is assembly-defective, it co-assembles with the wild-type dimer, forming capsids that are indistinguishable from normal capsids, although with weakened protein-protein bonding^27,31^. Circular dichroism, protein fluorescence and chromatographic studies did not reveal any structural differences between the wild-type and Cp149-Y132A^29^, and the compound NVR-010–001-E2 had essentially identical antiviral effects on wild-type and Y132A mutant capsids^28^. Also, both the wild-type and Y132A mutant render human hepatoma HepG2 cells susceptible to TNFα-induced apoptosis by interacting with receptor of activated protein kinase C1 (RACK1)^32^. These structural and functional similarities indicate that Cp149-Y132A is a reasonable model for testing and developing antiviral compounds^12,28,30,33^. Unfortunately, in many cases, unexpected cytotoxicity has seriously hampered the clinical use of other antiviral compounds^12,22,28,30,34,35^.

To identify drugs that can clear HBV infections, we here assess the effect of 978 FDA-approved compounds on HBV replication. Of 13 potential HBV replication inhibitors that we identify, only ciclopirox inhibits HBV capsid assembly and secretion of HBV DNA in infected cells in vitro and in mice. Ciclopirox is a synthetic antifungal agent that has been used to treat mycoses of the skin and nails for >20 years^36^. The crystal structure of HBV core protein complexed with ciclopirox shows that ciclopirox occupies only three of six possible binding sites in the hydrophobic pocket at the dimeric interface of HBV core protein due to movement of the F23 residue and proline-rich loop 6. It also synergizes with TDF and ETV to inhibit HBV replication. Thus, ciclopirox may serve as an add-on drug for HBV-infected patients who are treated with TDF or ETV.

## Results

### Effect of FDA-approved compounds on HBV replication

**Fig. 1:**
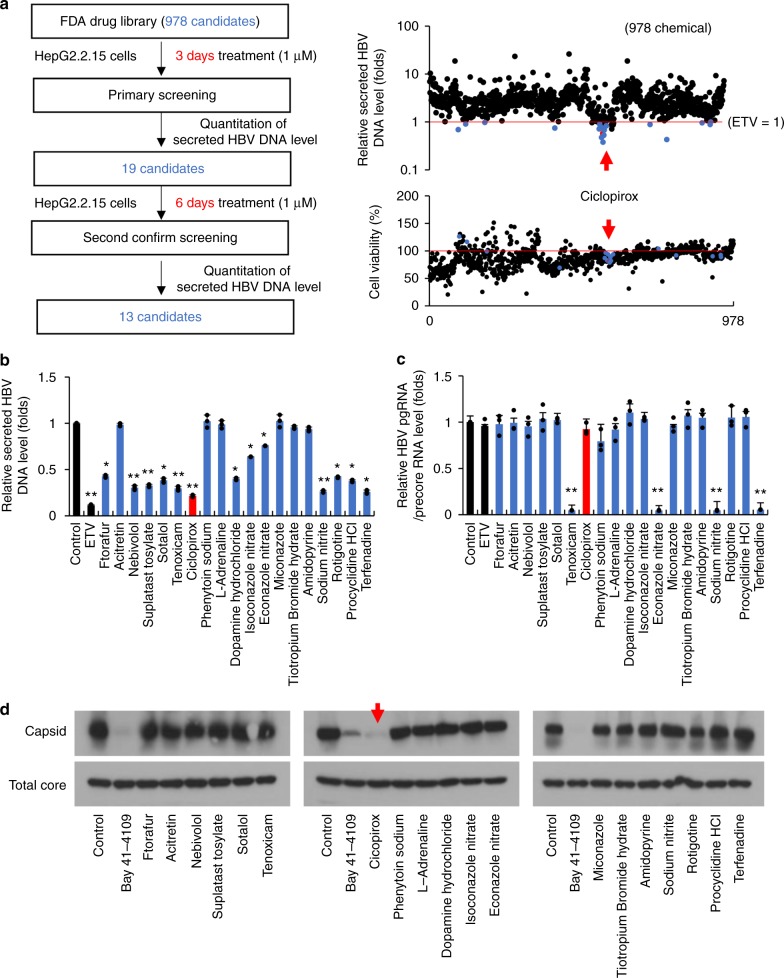
Effect of FDA-approved compounds on HBV replication. **a** Schematic depiction of the primary screening analysis. HepG2.2.15 cells, which were stably transfected with HBV replicons, were treated with 978 FDA-approved drugs (1 μM) for 3 days. Entecavir (ETV) (1 μM) served as positive control. The effects of the compounds on secretion of HBV DNA particles were assessed by quantitative PCR with an HBV DNA primer and probe set. **b** The 19 candidates that responded well in **a** were tested again in HepG2.2.15 cells for 6 days instead of 3 days. **c** The 19 candidates were assessed for their ability to inhibit HBV pgRNA and precoreRNA transcription in HepG2.2.15 cells. Quantitative RT-PCR was performed after 6 days of incubation. **d** Effect of the 19 candidates on intracellular HBV capsid assembly. Huh-7 cells were transfected with pCDNA3-Core and exposed after 12 h to the 19 candidates (1 μM) for 36 h. The cells were lysed and assembled HBV core particles were isolated by sucrose step gradient ultracentrifugation, resolved on 1% agarose gels, and subjected to immunoblot analysis with anti-HBV core antibody. The data shown in **b**–**d** are representative of three independent experiments and are expressed as means ± SDs. The error bars represent the ± SD. *p < 0.05; **p < 0.01, as determined by unpaired two-tailed Student’s *t*-tests. Source data are provided as a Source Data file

HepG2.2.15 cells are cells in which the ayw subtype (genotype D) HBV replicon is stably integrated^37^. These cells were exposed to 1 μM of each compound for 3 days in a high-throughput screen for their effects on HBV DNA secretion. Treatment with 1 μM ETV served as control. HBV DNA secretion was measured by quantitative PCR using an HBV DNA primer and probe set (Supplementary Table 1); after 3 days’ incubation with ETV, HBV DNA particle secretion was reduced by 42.1 ± 14.1%. Of the tested compounds, 19 were at least as effective as ETV (Supplementary Table 2). However, since the inhibitory effect of some of the 19 compounds varied considerably (Fig. 1a), we repeated the test with a longer treatment period (6 days). Thirteen compounds stably inhibited the secretion of HBV DNA (Fig. 1b). We then assessed by quantitative PCR how well these 19 compounds inhibited intracellular HBV transcription. Four compounds dramatically reduced intracellular HBV transcript levels (Fig. 1c). Finally, the effect of these 19 compounds on intracellular HBV capsid formation was analyzed. Only ciclopirox inhibited HBV capsid assembly (Fig. 1d). Collectively the results showed that ciclopirox inhibited HBV DNA secretion most potently (Fig. 1b), possibly by inhibiting HBV capsid assembly. Therefore we evaluated ciclopirox as an anti-HBV compound.

### Ciclopirox inhibits in vitro and intracellular HBV capsid assembly

**Fig. 2:**
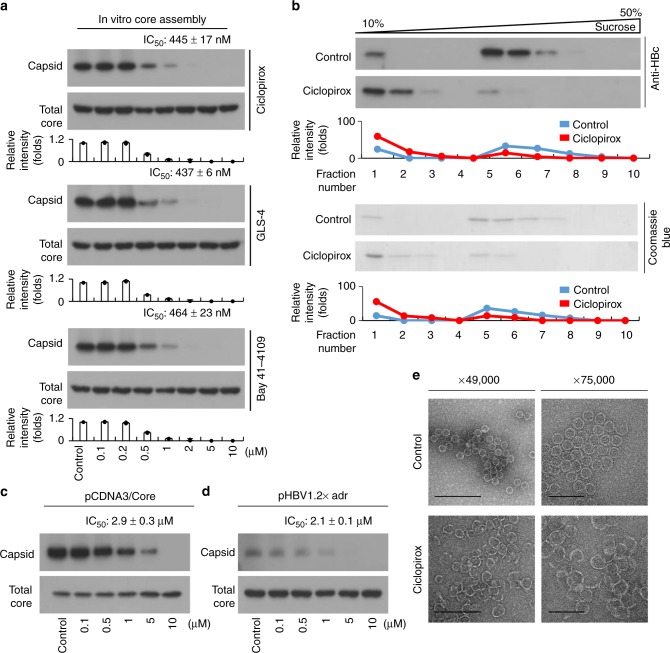
Ciclopirox inhibits in vitro and intracellular HBV capsid assembly. **a** Effects of ciclopirox, GLS-4, and Bay41–4109 on in vitro HBV capsid assembly. Various concentrations of ciclopirox, GLS-4, or Bay41–4109 were mixed with Cp149 in reaction buffer. After 1 h, the mixtures were subjected to **a** immunoblot analysis with anti-HBV core antibody. **b** Sucrose density gradient analysis of capsid assembly inhibition by ciclopirox. Ten fractions collected from top to bottom underwent immunoblot analysis. **c**, **d** Effect of ciclopirox on intracellular HBV capsid assembly. Huh-7 cells were transfected with (**c**) pCDNA3-Core or (**d**) pHBV1.2× and exposed after 12 h to the indicated concentrations of ciclopirox for 36 h. The cells were lysed and assembled HBV core particles were isolated by sucrose step gradient ultracentrifugation, resolved on 1% agarose gels, and subjected to immunoblot analysis with anti-HBV core antibody. **e** Electron micrographs of the effect of ciclopirox on in vitro HBV capsid assembly. The particles were negatively stained with 2% uranyl acetate. Scale bars = 200 nm and 100 nm. TEM magnifications, ×49,000 and ×75,000. Ciclopirox appeared to convert the *T* = 4 HBV capsid into *T* = 3 form (30 nm). The data shown in **a**–**e** are representative of three independent experiments and are expressed as mean ± SD. The error bars represent the ± SD. *p < 0.05; **p < 0.01, as determined by unpaired two-tailed Student’s *t*-tests. Source data are provided as a Source Data file

Of the selected compounds, only ciclopirox affected intracellular HBV capsid assembly at a concentration of 1 μM. To calculate the half maximal inhibitory concentration (IC_50_) of ciclopirox for in vitro HBV capsid assembly, Cp149, the truncated HBV core protein, was incubated with 0.1–10 μM ciclopirox in vitro and the mixtures were subjected to immunoblot analysis with an anti-HBV core antibody. The IC_50_ value of ciclopirox was 445 ± 17 nM (Fig. 2a). We also tested previously-discovered compounds such as GLS-4 and Bay41–4109 for inhibition of HBV capsid assembly. The IC_50_ values of GLS-4 and Bay41–4109 were 437 ± 6 nM and 464 ± 23 nM, respectively (Fig. 2a). In addition to assembly of HBV capsid, we also tested the ability of ciclopirox to disrupt assembled HBV capsids, but the efficiency was low (27 μM) compared to inhibition of HBV capsid assembly (Supplementary Fig. 1). The ability of ciclopirox to disrupt HBV capsid assembly in vitro was verified by sucrose density centrifugation; in the presence of ciclopirox, movement of the unassembled HBV core proteins (fraction numbers 1–3) into the assembled HBV core protein fraction (fraction numbers 5–8) was blocked (Fig. 2b).

We also measured the IC_50_ of ciclopirox for intracellular HBV capsid assembly. Huh-7 cells were transfected with pCDNA3-Core (an HBV core-expressing plasmid) and treated with various concentrations of ciclopirox for 36 h. Immunoblot analysis with an anti-HBV core antibody showed that ciclopirox inhibited HBV capsid assembly with an IC_50_ of 2.9 ± 0.3 μM. Total denatured HBV core protein was not affected (Fig. 2c). However, previous reports have shown that Bay41–4109 and GLS-4 reduce intracellular total core protein by inducing its degradation^38^. In our hands, total core protein was also decreased by ciclopirox, Bay41–4109 and GLS-4 if the treatment time was increased, although the extent of the degradation varied (Supplementary Fig. 2). In an analysis using Huh-7 cells transfected with the pHBV1.2× (subtype adr; genotype C) replicon, the IC_50_ for ciclopirox for incubation for 36 h was 2.1 ± 0.1 μM (Fig. 2d).

The structures generated by in vitro HBV capsid assembly of Cp149 with or without ciclopirox were then examined by EM. In the absence of ciclopirox, the 30-nm-diameter HBV core particles were formed. In the presence of ciclopirox, HBV capsid morphology was altered; the structures formed were larger with a more open shape (Fig. 2e). Thus, ciclopirox inhibited normal HBV capsid assembly in vitro.

### Overall structure of HBV core protein complexed with ciclopirox

**Fig. 3:**
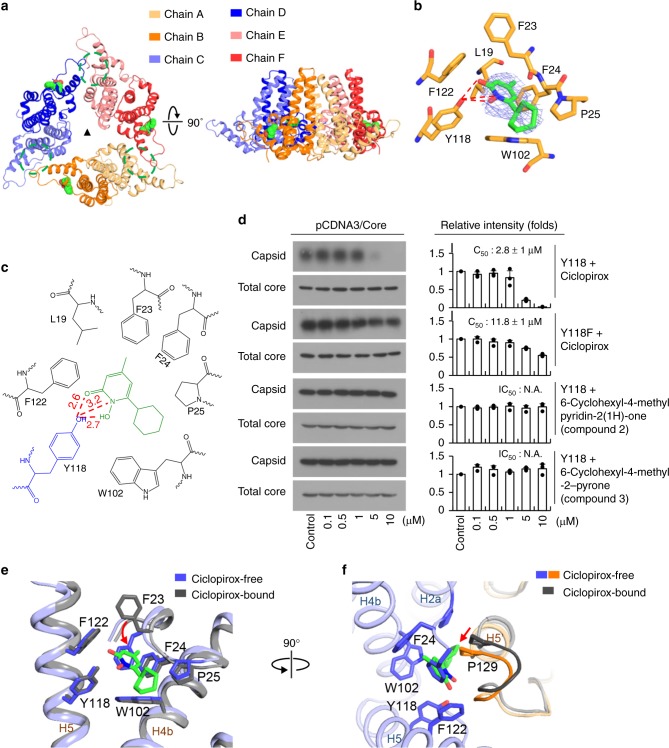
Characterization of the ciclopirox binding sites on HBV core protein. **a** Overall architecture of the hexamer in the asymmetric unit. The six protomers are colored light orange, orange, light blue, blue, pink, and red. The secondary structure of the protein was calculated using STRIDE^62^. Ciclopirox is shown as a space-filling model. The binding sites at the dimer-dimer interfaces that are not bound by ciclopirox are highlighted by dashed green circles. The black triangle indicates the icosahedral threefold axis of the HBV capsid. Top view (left panel) and side view (right panel). **b** The ciclopirox binding site in chain B. The view is the same as in the right panel of **a**. The residues involved in ciclopirox binding are in orange. The simulated annealing omit map superimposed on the refined ciclopirox model was contoured at the 2.5σ level. **c** Schematic depiction of the HBV core protein-ciclopirox interaction. The residues involved in ciclopirox binding are shown. Y118 and ciclopirox are in blue and green, respectively. **d** Effect of mutation of Y118 to phenylalanine (Y118F) and chemical modifications of ciclopirox on intracellular HBV capsid assembly. **e** Comparison of the ciclopirox-bound and -free sites of chains (**b**, **c**). The two binding sites are aligned and the residues involved in ciclopirox binding are represented as sticks. The leucine at 19 is omitted for clarity. The view is the same as in **b**. The rotation of F23 is marked by the red arrow. **f** Movement of the proline-rich loop 6. The ciclopirox-free pocket is shown, and ciclopirox and loop 6 of the ciclopirox-bound protomer are superimposed on it. The movement of loop 6 is shown by the red arrow. This view is rotated by 90 degrees along the vertical axis from (**e**). The data in **d** are representative of three independent experiments and are expressed as mean ± SD. The error bars represent the ± SD. N.A., not available. *p < 0.05; **p < 0.01, as determined by unpaired two-tailed Student’s *t*-tests. Source data are provided as a Source Data file

In order to understand the interaction between HBV core protein and ciclopirox at the molecular level, we purified and crystallized Cp149-Y132A in the presence of ciclopirox. A 2.3-Å resolution dataset was collected, and was subsequently refined to *R*_work_ of 25.8% and *R*_free_ of 30.4%, with good geometry (Table 1). As in the structures of Cp149-Y132A complexed with other assembly modulators^12,28,30^, the complex with ciclopirox consisted of an asymmetric unit made up of a closed trimer of CD dimers, positioned in a similar fashion around the icosahedral threefold axis of the wild-type HBV capsid (Fig. 3a)^25^. Structure alignments of the wild-type and Y132A mutant HBV core proteins showed that the central cores containing the putative binding sites for ciclopirox (residues 1–62 and 94–142) were almost identical, with an overall Cα rms difference of 0.9 Å, although the spike regions containing the upper bundles of four α-helices and the contact regions at the dimeric interface differed (Supplementary Fig. 3). Cp149-Y132A was arranged in head-to-head mode in the crystal packing (Supplementary Fig. 4). Since the three dimers in the hexamer are all fundamentally the same, with a Cα rmsd of 0.2 Å, it appears that this “trimer-of-dimers” is a structurally stable but abnormal intermediate in HBV capsid assembly that arises under our crystallization conditions (Supplementary Fig. 5a). Structural alignment of the six individual protomers showed that while the HBV core regions aligned relatively well, there were large structural changes in two areas, namely, in the spike and the contact regions (Supplementary Fig. 5b). Based on these structural differences, the protomers forming the hexamer can be divided into two groups, each of which forms a highly asymmetric dimer. When the dimers from previously reported structures were superimposed on ours, there was poor alignment in the spike and contact regions (Supplementary Fig. 6). This suggests that our ciclopirox-complex has a completely novel fold.

**Table 1:** Data collection and refinement statistics

	Ciclopirox bound Cp149-Y132A
*Data collection*	
Space group	C2
Cell dimensions	
*a*, *b*, *c* (Å)	153.9, 88.5, 99.1
*α*, *β*, *γ* (°)	90.0, 121.1, 90.0
Resolution (Å)	50.0–2.3 (2.38–2.30)*
*R*_sym_ or *R*_merge_	0.049 (0.292)*
*I* / σ*I*	30.1 (3.1)*
Completeness (%)	99.2 (99.3)*
Redundancy	3.5 (3.3)*
*Refinement*	
Resolution (Å)	50.0–2.3
No. reflections	47,285/2382
*R*_work_ / *R*_free_	25.8/30.4
*No. atoms*	
Protein	6609
Ligand/ion	45
Water	292
*B*-factors (Å^2^)	
Protein	79.9
Ligand/ion	118.6
Water	82.8
*R.m.s. deviations*	
Bond lengths (Å)	0.004
Bond angles (°)	1.367

^*^Values in parentheses are for highest-resolution shell

### Characterization of ciclopirox binding sites on HBV core protein

There are six binding sites for assembly modulators in the HBV core protein hexamer^12,28,30^; in all cases, one molecule of modulator binds to each of the three dimer-dimer interfaces, while three other modulator molecules nestle in similar hydrophobic pockets formed by the dimers and their equivalents formed by symmetry-related HBV core proteins. Surprisingly, in our crystal structure ciclopirox occupied only the latter pockets (Fig. 3a). This half occupancy by ciclopirox is probably not due to the presence of insufficient ciclopirox during crystallization because an approximately two-fold molar excess of ciclopirox was used in an effort to saturate the protein. Comparisons of the ciclopirox-bound and other assembly modulator-bound forms of Cp149-Y132A revealed that ciclopirox adopted an overall similar binding mode but with subtle but significant differences, and therefore interacted in new ways with HBV core protein. The binding pocket was formed by hydrophobic residues L19, F23, F24, P25, Y118, F122, and W102, with extensive van der Waals interactions between ciclopirox and these residues (Fig. 3b, c and Supplementary Fig. 7). Y118 in particular played a key role in securing the ciclopirox, by forming strong hydrogen bonds and salt bridges with a carbonyl oxygen, a nitrogen, and a hydroxyl group of the pyridine ring of ciclopirox. To confirm the proposed role of Y118 in the inhibition of normal HBV capsid assembly by ciclopirox, we performed a site-directed mutagenesis experiment in which Y118 was mutated to phenylalanine (Y118F) to disrupt the hydrogen bond interactions with ciclopirox. To analyze the responses of the Y118F mutant to ciclopirox, in vitro intracellular HBV capsid assembly was measured after treatment with various concentrations of ciclopirox using Huh-7 cells transfected with pCDNA3-Core Y118F (Fig. 3d). We found that the Y118F mutant had similar levels of total expression as the wild-type control. However, normal HBV capsid assembly in the Y118F mutant was inhibited by ciclopirox with an IC_50_ of 11.8 ± 2 μM, an approximately 4-fold reduction in sensitivity compared with the wild-type. In addition, modification of the binding region of ciclopirox (6-cyclohexyl-4-methyl pyridin-2(1 H)-one: removal of the hydroxy group of the pyridine core skeleton; 6-cyclohexyl-4-methyl −2-pyrone: changing the pyridone core skeleton to a pyrone moiety) for HBV core protein blocked its HBV capsid assembly inhibitory activity in vivo (Fig. 3d) and in vitro (Supplementary Fig. 8). Based on our structure and the mutational analysis we conclude that Y118 has an important role in stabilizing the conformation of ciclopirox in the hydrophobic pocket, as well as in the response to ciclopirox in terms of inhibition of normal HBV capsid assembly.

When the hydrophobic pocket to which ciclopirox binds was compared to the hydrophobic pocket at the dimer-dimer interfaces to which ciclopirox does not bind, it became clear why the latter pocket cannot bind to ciclopirox despite the fact that the two pockets have very similar shapes and share many of the same residues. First, the absence of binding of ciclopirox causes F23 to rotate by 142 degrees (Fig. 3e) and to place its phenyl ring into the pocket. In this conformation, the phenyl ring of F23 directly collides with the pyridine ring of ciclopirox, thereby disallowing ciclopirox to bind to the pocket. It is noteworthy that the conformation of the F23 residue in previous structures is very similar to that in the ciclopirox-free pocket^12,28,30^ (Supplementary Fig. 9). This suggests that, to avoid colliding with F23 the binding sites of other modulators shift towards the periphery. The second reason why ciclopirox does not bind to the non-binding pocket is that the proline-rich loop (loop 6, residues 128–134) surrounding the dimer-dimer interface moves ~2 Å into the binding pocket when ciclopirox is absent; if ciclopirox enters the non-binding pocket, its pyridine ring may clash sterically with P129 of the loop (Fig. 3f). By contrast, with the other assembly modulators, loop 6 appears to move outwards to create enough space for binding (Supplementary Fig. 10). These findings suggest that when ciclopirox does not bind to the HBV core protein both F23 and loop 6 undergo rearrangements that reduce the total volume of the dimer-dimer interface pocket, blocking the entry of ciclopirox. These rearrangements may also help to stabilize the empty hydrophobic pocket.

### Ciclopirox inhibits HBV replication

**Fig. 4:**
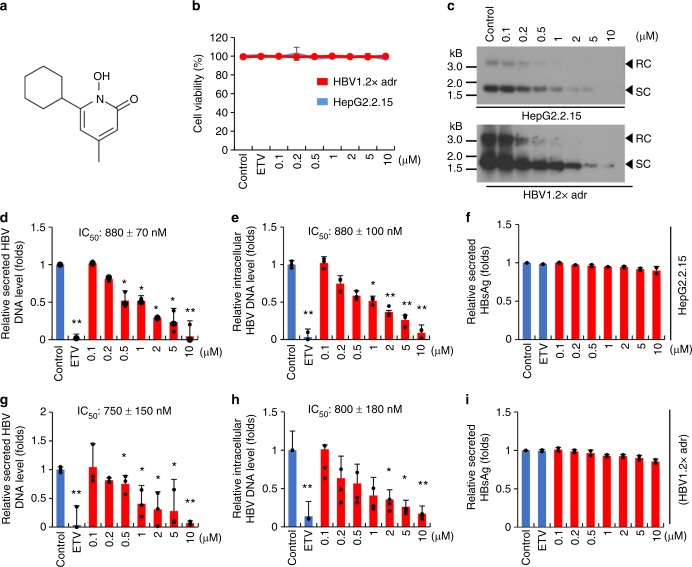
Ciclopirox inhibits HBV replication. **a** Chemical structure of ciclopirox. **b** HepG2.2.15 cells (blue), and HepG2 cells transfected with pHBV1.2× (red) were treated with various concentrations (0.1–10 μM) of ciclopirox for 6 days and assayed for cytotoxicity. **c** Intracellular HBV DNA was detected by southern blot analysis. **d**–**i** Secreted HBV DNA (**d**, **g**, respectively) and intracellular HBV DNA (**e**, **h**, respectively) were measured by quantitative PCR. Secreted HBsAg (**f**, **i** respectively) was quantified by ELISA. The data in **b**–**i** are representative of two or three independent experiments and are expressed as mean ± SD. The error bars represent the ± SD. *p *<* 0.05; **p < 0.01, by unpaired two-tailed Student’s *t*-tests. Source data are provided as a Source Data file

Ciclopirox is the ethanolamine salt of 6-cyclohexyl-1-hydroxy-4-methyl-2(1 H)-pyridone^39^ (Fig. 4a). In Fig. 1b, we showed that when HepG2.2.15 cells were treated with 1 μM ciclopirox, secreted levels of HBV DNA dropped markedly. We also showed that ciclopirox inhibited normal HBV capsid assembly (Fig. 2). We have confirmed and expanded these findings in additional experiments with HepG2.2.15 and HepG2 cells transfected with pHBV1.2× (Fig. 4b–i). When cells of these two cell lines were treated with 0.1–10 μM ciclopirox for 6 days there was no significant cytotoxicity (Fig. 4b), in contrast with a previous report that ciclopirox reduced HepG2 cell viability, even though this cell line was less sensitive to ciclopirox than other cancer cell lines in the data of ref. ^41^. We showed that the lack of inhibition in our case was due to the high iron level in fetal bovine serum (FBS), because lowering the percentage FBS, and deferoxamine (iron chelator) treatment, reduced cell viability (Supplementary Fig. 11). Southern blot analyses showed that ciclopirox reduced intracellular HBV DNA levels in a dose-dependent manner (Fig. 4c). Quantitative PCR analyses showed that the IC_50_ values of ciclopirox for HBV DNA secretion by HepG2.2.15 and by the transfected cells were 880 ± 70 nM (Fig. 4d) and 750 ± 150 nM (Fig. 4g), respectively. Quantitative PCR analyses also showed that the IC_50_ values for intracellular HBV DNA were 800 ± 100 nM (Fig. 4e) and 800 ± 180 nM (Fig. 4h), respectively. Thus, ciclopirox inhibited HBV DNA production in both cell lines. At the same time, ciclopirox had no effect on HBsAg production (Fig. 4f, i) or HBV transcripts levels (Supplementary Fig. 12). We conclude that ciclopirox inhibits the secretion of particles containing viral DNA by inhibiting HBV capsid assembly, not as the result of cytotoxicity or by limiting HBV transcription or the production of HBV protein.

### Ciclopirox inhibits HBV replication in in vitro infection models

**Fig. 5:**
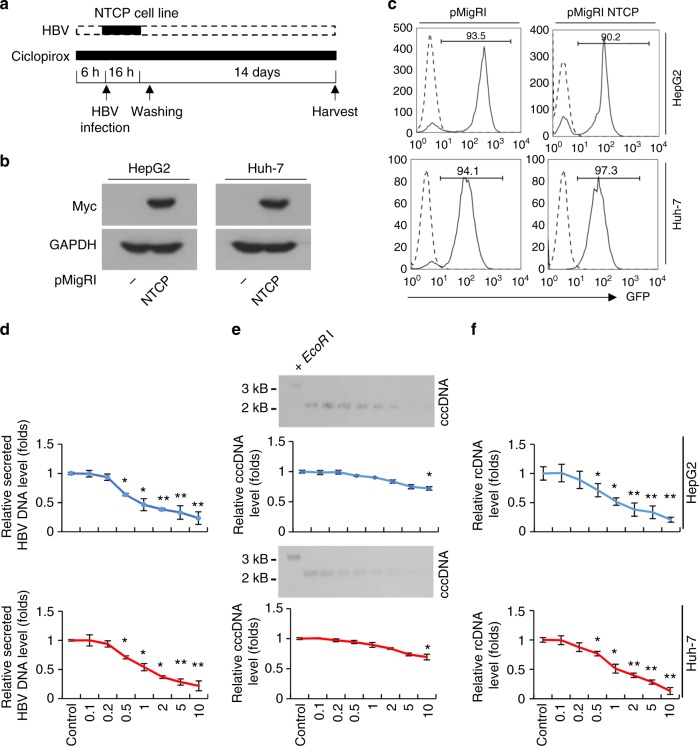
Ciclopirox inhibits HBV replication in an in vitro HBV infection model. **a** Schematic depiction of the experiment. HepG2-NTCP and Huh-7-NTCP cells were treated with ciclopirox for 6 h and exposed to HBV for 16 h. After washing, the cells were treated daily with ciclopirox and cultured for another 14 days. **b** NTCP protein expression analyzed by immunoblot analysis. **c** GFP protein expression analyzed by flow cytometry. **d**–**f** Quantitative PCR with an HBV DNA primer and probe set was used to measure secreted HBV DNA (**d**), intracellular cccDNA (**e**), and intracellular HBV rcDNA (**f**). Intracellular cccDNA was also detected by southern blot analysis (**e**). The data in **b**–**f** are representative of three independent experiments and are expressed as mean ± SD. The error bars represent the ± SD. *p < 0.05; **p *<* 0.01, by unpaired two-tailed Student’s *t*-tests. Source data are provided as a Source Data file

Recently, HBV preS1 was found to mediate HBV entry into cells by binding to the sodium-dependent taurocholate cotransporter polypeptide (NTCP). This led to the development of in vitro HBV infection models, namely, NTCP-expressing HepG2 and Huh-7 cells. Expression of this protein causes these cells, which are normally resistant to infection with HBV particles, to become highly susceptible to infection^40^. We used similar models to determine the effect of ciclopirox on in vivo HBV replication (Fig. 5a). HepG2 and Huh-7 cells permanently expressing NTCP were generated by infecting them with an NTCP-encoding recombinant retrovirus and isolating GFP-expressing cells. NTCP expression in the resulting clones was confirmed by immunoblot analysis with anti-Myc antibody (Fig. 5b) and by detecting GFP expression by flow cytometry (Fig. 5c). When these cell lines were treated with ciclopirox, secretion of HBV DNA (Fig. 5d), cccDNA levels (Fig. 5e), and intracellular relaxed circular DNA levels (Fig. 5f) dropped markedly. However, our data did not indicate specific regulation of cccDNA by ciclopirox because TDF also reduced cccDNA levels (Supplementary Fig. 13). In this analysis, the cccDNA obtained was treated with *EcoR*I to identify linearized cccDNA (Fig. 5e and Supplementary Fig. 14). In summary, ciclopirox blocks HBV replication in these model systems, indicating that it may be useful for developing new anti-HBV agents.

### Effects of treatments combining ciclopirox with TDF or ETV

**Fig. 6:**
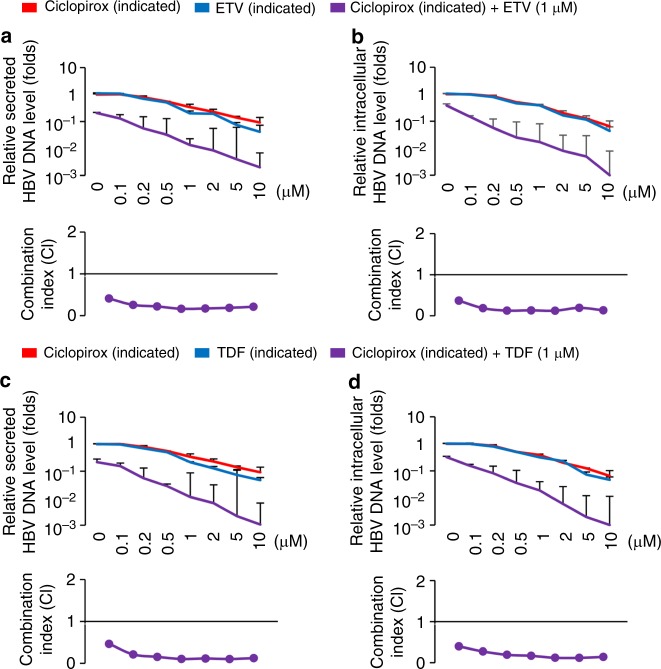
Effect of combined ciclopirox and TDF and ETV on replication of HepG2.2.15 cells. HepG2.2.15 cells were treated with 1 μM TDF or ETV and various concentrations (0.1–10 μM) of ciclopirox. **a**–**d** Secreted HBV DNA (**a**, **c**, respectively) and intracellular HBV DNA (**b**, **d**, respectively) were measured by quantitative PCR. Combination indices (CI) were analyzed by the Chou-Talalay method. The data in (**a**–**d**) are representative of three independent experiments and are expressed as mean ± SD. The error bars represent the ± SD. *p *<* 0.05; **p < 0.01, by unpaired two-tailed Student’s *t*-tests. Source data are provided as a Source Data file

Next-generation therapies for chronic HBV infection are likely to include combinations of established and novel drugs^7,41^. Several studies have investigated the ability of TDF and ETV in combination to inhibit HBV replication^41,42^. We therefore asked whether combining ciclopirox with TDF or ETV improved the anti-HBV effect. Indeed, when HepG2.2.15 cells were treated with 1 μM ETV (Fig. 6a, b) or TDF (Fig. 6c, d) and various ciclopirox concentrations (0.1–10 μM), we observed synergistic inhibition of HBV DNA secretion (Fig. 6a, c) and intracellular HBV DNA levels (Fig. 6b, d), based on analysis of the data by the Chou-Talalay method. We conclude that ciclopirox synergizes with established drugs such as TDF and ETV in inhibiting HBV replication.

### Antiviral effect of ciclopirox in a mouse model of HBV infection

Before assessing the antiviral effect of ciclopirox on HBV replication in a mouse model, the oral toxicity of ciclopirox for mice was tested. BALB/c male mice (5-weeks-old) were treated orally with ciclopirox at concentrations of 0, 1, 5 mg per kg, daily for 4 weeks. Five mice were included in each group. Mortality was monitored daily, and bodyweight was measured every other day. On day 28, blood was taken from the orbital sinus, and white blood cells, red blood cells and platelets were counted. Blood urea nitrogen, creatinine, glucose, total bilirubin, total cholesterol, triglyceride, albumin, total protein, aspartate aminotransferase, alanine aminotransferase (ALT), sodium, potassium, and chloride were measured. Urinalysis was performed including urobilinogen, glucose, bilirubin, ketone, specific gravity, blood, pH, protein, nitrate, and leukocytes. No significant oral toxicity was noted at either concentration of ciclopirox (Supplementary Table 3).

**Fig. 7:**
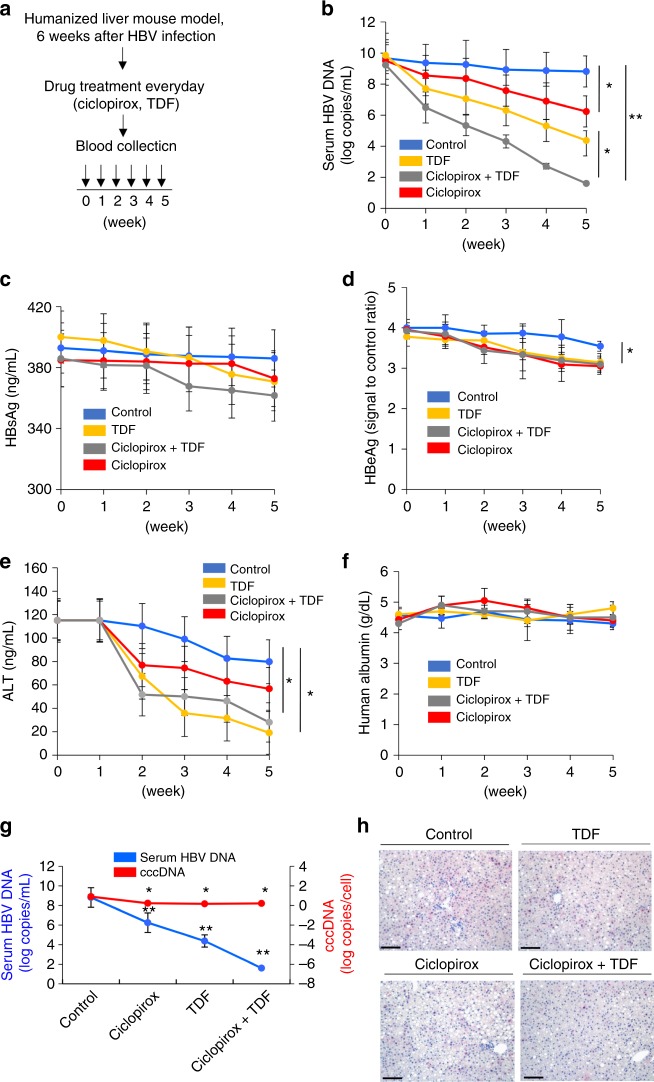
Antiviral activity of ciclopirox in a humanized liver mouse model of HBV infection. **a** Schematic depiction of the experiment. PXB mice (*n* *=* 3 per group) were injected via the tail vein with HBV virions (5 ×  10^7^ copies per mouse, genotype D). After 6 weeks, the mice were treated orally with TDF and/or ciclopirox (both 5 mg per kg, daily), and serum samples were taken from the orbital sinus every week. **b** Serum HBV genomic DNA levels measured by quantitative PCR. **c** Serum HBsAg levels determined by ELISA. **d** Serum HBeAg levels determined by ELISA. **e** Serum ALT levels determined by ELISA. **f** Human albumin levels determined by ELISA. **g** Intrahepatic cccDNA was determined in nucleic acid extracted from liver specimens obtained after 35 days of treatment. **h** Immunohistochemical analysis of hepatitis B core antigen (HBcAg) in liver sections obtained after 35 days of treatment (×200 magnification). The data in **b**–**g** are representative of three independent experiments, and are expressed as means ± SDs. The error bars represent the ± SD. Scale bars, 100 μm.*p < 0.05; **p *<* 0.01, by unpaired two-tailed Student’s *t*-tests. Source data are provided as a Source Data file

Then the antiviral effect of ciclopirox on HBV replication in a humanized liver mouse model was assessed. PXB (human liver-chimeric uPA/SCID) mice were injected intravenously with HBV virion, and after 6 weeks the mice were treated daily with ciclopirox and/or TDF (Fig. 7a). By week 5 of treatment, ciclopirox had lowered serum HBV DNA levels significantly and these effects were enhanced when it was combined with TDF (Fig. 7b). None of these treatments altered serum HBsAg (Fig. 7c). By week 5, ciclopirox or TDF treatment had significantly decreased HBeAg levels compared to the control (Fig. 7d). Serum ALT levels also decreased in response to TDF and/or ciclopirox (Fig. 7e). Human albumin levels did not change significantly compared to baseline in any of the groups, implying that there was no significant drug-induced liver injury (Fig. 7f). By week 5, both ciclopirox and TDF significantly reduced the amount of intrahepatic cccDNA (Fig. 7g). However, the reduction of cccDNA caused by ciclopirox was small compared to the decline of serum HBV DNA levels. Ciclopirox also significantly reduced HBV core protein levels in the liver with/without TDF (Fig. 7h). The antiviral effect of oral ciclopirox on HBV replication in a hydrodynamic injection mouse model was also assessed. The tail veins of C57BL/6 mice were injected hydrodynamically with the HBV replicative plasmid, pAAV-HBV1.2× (genotype D), and the mice were treated daily with ciclopirox and/or TDF^43^. By day 5, ciclopirox (5 mg per kg) and/or TDF (5 mg per kg) had reduced HBV core protein levels in the liver and significantly lowered serum HBV DNA levels (Supplementary Fig. 15).

## Discussion

Currently, pegIFNα2a and NAs such as TDF and ETV are the recommended first-line agents for suppressing HBV viral replication in chronically infected patients. However, loss of HBsAg is rarely achieved, and more effective therapies are needed. Direct-acting antivirals (DAAs) are medications that target specific steps in the hepatitis C virus (HCV) life cycle. In phase III clinical trials targeting multiple steps of the HCV life cycle, DAAs caused a sustained virological response in >90–95% of HCV-infected patients^44^. However, in HBV-infected patients the frequency with which DAA monotherapies that target the HBV polymerase induce HBsAg loss is very low^45^. Thus, to completely eradicate HBV infection it may be necessary to target multiple steps in the HBV life cycle, thereby preventing or strongly reducing HBV replication in infected hepatocytes and blocking de novo cccDNA formation. Several steps of the HBV life cycle have been targeted to date; as a result, multiple HBV DAA candidates that inhibit HBV polymerase, the entry receptor, HBV capsid assembly, cccDNA formation, or HBV transcription are in the clinical or preclinical stage of drug development. For example, the entry inhibitor Myrcludex-B is currently being evaluated in phase IIa clinical trials. However, although this drug prevents HBV from spreading from infected hepatocytes, it must be delivered by intravenous injection and is thus not suitable for general HBV-infected patients^46^. Another example of an HBV DAA is the HBV capsid assembly inhibitor, NVR 3–778, which is currently being evaluated in phase II clinical trials. This inhibitor blocks the formation of replication-competent HBV particles and may be suitable as an add-on treatment with established NAs^14^.

The present study shows that ciclopirox acts as an anti-HBV agent by inhibiting normal HBV capsid assembly. However, minor effects of ciclopirox on HBV replication cannot be entirely excluded because it affects the regulation of several intracellular processes that account for most of the anti-cancer actions of ciclopirox^47–50^. A previous report has claimed that ciclopirox inhibits HIV-1 replication through control of HIV-1 transcription^51^, but our data clearly show that ciclopirox does not affect HBV transcription (Supplementary Fig. 12).

A first-in-human phase I study recently tested the efficacy and safety of ciclopirox when orally administered for the treatment of hematological malignancies. When patients were treated once daily with >10 mg per m^2^ ciclopirox olamine®, it suppressed anti-apoptotic gene expression in peripheral blood cells. Dose-limiting gastrointestinal toxicities were observed in patients who received 80 mg per m^2^ four times daily but not in those who received 40 mg per m^2^ once daily^52^ (~1 mg per kg for humans, which is equivalent to 12.3 mg per kg for mice^53^). Since our study showed that ciclopirox had significant anti-HBV activity at 5 mg per kg in the mouse system, the preclinical ciclopirox data suggest that this drug may be a safe add-on to the currently recommended oral treatment regimens even extensive oral test for anti-HBV activity is needed. This view is supported by the most striking finding of this study, namely that ciclopirox reduces HBV particle secretion synergistically with TDF and ETV. Since this synergistic effect may reflect the fact that multiple steps in the HBV life cycle (i.e., HBV polymerase and HBV capsid assembly) are targeted by the combination treatment, it supports the notion that targeting multiple HBV life cycle steps improves HBV treatment efficacy. The suitability of ciclopirox as an HBV therapy will be assessed in future studies.

The atomic structure of ciclopirox-bound HBV core protein has offered several insights into how this compound binds to HBV core protein. First, we found that ciclopirox binds to only three of the six sites to which other assembly modulators bind (Fig. 3a). Second, in addition to extensive hydrophobic interactions, Y118 forms essential hydrogen bonds and salt bridges with ciclopirox to anchor it in the binding pocket. Our data also confirmed that disruption of these hydrogen bonds by mutation of tyrosine to phenylalanine at 118 (Y118F) significantly reduced ciclopirox-mediated inhibition of normal HBV capsid assembly (Fig. 3b–d). It is noteworthy that in the previously reported structures of Cp149-Y132A complexed with other assembly modulators, Y118 of the HBV core protein formed only hydrophobic interactions, not hydrogen bonds^12,28,30^. Third, the mode of binding of ciclopirox differs significantly from those of other HBV capsid modulators, including NVR-010–001-E2^28^, 4-methyl HAP^30^, SBA_R01^12^, and HAP_R01^12,22^. Specifically, when ciclopirox binds to HBV core protein a major conformational change occurs; F23 undergoes a phenyl ring rotation (Fig. 3e) that causes the ring to move from an inwardly pointing position in the pocket (which blocks inhibitor binding) to an outwardly pointing position (which allows inhibitor binding). This rotation allows ciclopirox to wedge itself deep into the HBV core protein (Supplementary Fig. 9). Since ciclopirox is a relatively low MW compound, this deep binding seems to promote hydrophobic interactions and to prevent escape back into the solvent. Meanwhile, in the ciclopirox-free pocket, the F23 side chain forms hydrophobic bonds with neighboring residues, thereby preventing entry of ciclopirox into the pocket (Fig. 3e). Moreover, despite the large conformational change of F23, the ciclopirox-bound pocket is also stabilized because its pyridine ring compensates for the loss of the phenyl ring of F23 (Fig. 3e). Interestingly, these observations are reminiscent of the behavior of the F23A mutant, which cannot form the HBV capsid^26^.

Our observations suggest that ciclopirox may bind to the HBV core protein as follows. First, it preferentially binds to half of the possible binding pockets either before or after HBV capsid formation; this leads to essential interdimer interactions and causes structural changes of the subunit (Supplementary Fig. 16). Highly asymmetrical dimers may thus be formed, which in turn lead to the formation of irregular or malformed HBV capsids that produce fewer HBV virions. Unfortunately, the current data do not allow us to determine why ciclopirox has a unique binding site preference, or how ciclopirox and TDF and ETV synergize in inhibiting HBV replication.

## Methods

### Cell lines

HepG2 (hepatoblastoma cell line, ATCC, HB-8065), Huh-7 (hepatocellular carcinoma cell line, Korea cell line bank, 60104), HepG2.2.15 (HBV-producing cells)^37^, HepG2-NTCP, and Huh-7-NTCP cells were cultured at 37 °C in 5% CO2 in Dulbecco′s Modified Eagle′s Medium with 10% FBS and 100 U penicillin with 0.1 mg per mL streptomycin. The same conditions were used to culture HepG2 and Huh-7 cells that were transfected with pHBV1.2× (genotype D). All maintained cells tested by PCR were mycoplasma-negative.

### Quantitative PCR to measure intracellular HBV DNA and cccDNA

Cells were treated with the indicated compound(s), after which the HBV DNA that had been secreted into the medium, and intracellular HBV DNA and cccDNA, were extracted, purified and quantified by quantitative PCR. To isolate the secreted HBV DNA, culture media were collected and centrifuged at 15,000 × *g* to remove debris. The medium was diluted 1:1 with phosphate-buffered saline (PBS), and 1 M NaOH solution was added to a final concentration of 0.1 M. The mixture was incubated at 37 °C for 1 h. Protein was denatured by adding 2 M Tris-HCl (pH 7.5) solution to a final concentration of 0.2 M and incubating the mixture at 98 °C for 5 min. The protein precipitate was removed by centrifugation at 15,000 × *g*, and the supernatant was subjected to PCR with an HBV DNA primer and probe set (Supplementary Table 1). HBV DNA in the sera of mice infected with the replicative HBV plasmid pAAV-HBV1.2× (genotype C) was also measured by this method. To isolate intracellular HBV DNA, the cells were lysed on ice with lysis buffer (50 mM Tris-HCl, pH 7.5, 1 mM EDTA, 1% NP40) for 10 min. After removing cell debris and nuclei by centrifugation at 15,000 × *g*, the supernatant was treated with micrococcal nuclease (0.25 U per μL, M0247, New England Biolabs) at 37 °C for 1 h. The micrococcal nuclease was then inactivated by adding EGTA to a final concentration of 10 mM. Thereafter intracellular HBV DNA was extracted with a G-spin^TM^ total kit (17046, Intron). The isolated intracellular HBV DNA was subjected to PCR with an HBV DNA primer and probe set (Supplementary Table 1). cccDNA was isolated by the Hirt extraction method^54^. Briefly, the cells were lysed in SDS lysis buffer (50 mM Tris-HCl, pH 8.0, 10 mM EDTA, 150 mM NaCl, 1% SDS), and the lysates were mixed with KCl to a final concentration of 0.5 M. After incubation at 4 °C for 30 min, each lysate was centrifuged and the supernatant was extracted three times with phenol and once with chloroform. The DNA was then recovered by ethanol precipitation, and treated with T5 nuclease (10 U per μg for 0.5 h, M0363, New England Biolabs), and the enzyme was subsequently heat-denatured at 70 °C. The isolated cccDNA was subjected to PCR with a cccDNA primer and probe set (Supplementary Table 1). Quantification of cccDNA in liver tissue of HBV-infected PXB mice was also measured by this primer and probe set.

### Southern blot analysis for detecting HBV DNA

HBV DNA was separated on a 1% agarose gel, blotted onto a nylon membrane (Hybond N + , RPN203B, GE Healthcare), and hybridized with a biotin-labeled full length HBV fragment prepared with the BioPrime™ DNA labeling system (18094011, ThermoFisher).

### Infection of HepG2-NTCP and Huh-7-NTCP cells with HBV

HepG2-NTCP and Huh-7-NTCP cells were cultured in 12-well plates with the indicated compound(s) for 4 h, after which the cells were infected with HBV particles (10^7^ viral genome equivalent per mL, genotype D) and treated with the indicated compound(s) for another 16 h. After removing the virus-containing medium, the cells were cultured in the presence of the indicated compound(s) for another 14 days.

### Quantitative RT-PCR for measuring HBV transcription

Quantitative RT-PCR with primers that detect three HBV regions (R1, R2, and R3, Supplementary Table 1) was used to measure HBV transcription. This RT-PCR detects pgRNA and precore RNA (3.5 kB), two HBV surface-encoding RNAs (2.5 kB and 2.1 kB), and HBx-encoding RNA (0.7 kB)^55^. PCR conditions were 95 °C for 10 min initially, then 50 cycles of 95 °C for 30 s, 60 °C for 60 s and 72 °C for 30 s, and finally 1 cycle of 95 °C for 60 s, 55 °C for 30 s and 95 °C for 30 s.

### Analysis of intracellular HBV capsid assembly

Huh-7 cells were seeded in six-well plates at a density of 5 × 10^5^ cells per well and transfected with pHBV1.2×, pCDNA3-Core (genotype C), or pCDNA3-Core Y118F plasmid DNA. After 12 h, the cells were incubated with the indicated compound(s) for 36 h. They were then lysed on ice with lysis buffer (1% NP40) for 10 min, and cell debris and nuclei were removed by centrifugation at 15,000 × *g*. The supernatant was laid on a sucrose gradient composed of 1 mL of 40% sucrose (wt per vol) and 1.5 mL of 20% sucrose in 1 × PBS, and the HBV capsids were sedimented by centrifugation at 400,000 × *g* and 20 °C for 8 h. The pellets were resuspended in 50 μL of 1 × PBS and sonicated (1 s per stroke × 3 times), and samples were separated on 1% agarose gels. The gels were transferred onto nitrocellulose membranes by capillary transfer in 10 × SSC, and HBV core particles were detected by immunoblot analysis using anti-HBV core antibody (1:2000, B0586, Dako).

### Expression and purification of HBV core protein

Cp149 (amino acids 1–149) is a truncated form of the HBV core protein. It was expressed in *Escherichia coli* and the expressed protein was purified^56^. The gene encoding Cp149-Y132A with a C-terminal thrombin cleavage site was synthesized and optimized for expression in bacterial cells (Gene Universal). The gene was cloned between the *Nde*I and *BamH*I sites in pET23a vector containing a hexahistidine tag. Recombinant Cp149-Y132A was expressed in *E. coli* BL21 (DE3) cells that were cultured in LB medium at 37 °C. When the culture reached OD600 of 0.7–0.8, expression was induced with 0.5 mM isopropyl-β-d-thiogalactopyranoside, and the culture was cooled to 16 °C. The cells were harvested after 16 h, flash-frozen in liquid nitrogen, and stored at −80 °C. To purify Cp149-Y132A, thawed cells were resuspended in lysis buffer (20 mM Tris-HCl pH 9.0, 200 mM NaCl, 10 mM imidazole, 10 ug per mL DNaseI, 1 mM phenylmethylsulfonyl fluoride) and sonicated. The supernatant was loaded onto Ni-NTA affinity resin and the protein was eluted with a gradient of 20 to 500 mM imidazole in lysis buffer. After removing the histidine tag with thrombin, the protein was further purified by HiTrap Q anion exchange chromatography (17115401, GE Healthcare).

### Immunoblot analysis of HBV capsid assembly in vitro

To determine effects on Cp149 assembly in vitro, compounds were added to Cp149-containing suspensions. The final concentration of Cp149 was 1 mg per mL and the compounds were added at 0.1–10 μM. The suspensions were then mixed with reaction buffer (150 mM HEPES, pH 7.5, 15 mM NaCl) added at a ratio of 2:1. In vitro assembly was allowed to progress at 37 °C for 1 h and the HBV capsids were detected by immunoblot analysis using anti-HBV core antibody (1:2000, B0586, Dako). Uncropped and unprocessed scans of the most important blots are provided in the Source Data file.

### Sucrose density gradient analysis of HBV capsid assembly in vitro

Cp149 structures formed by assembly in the presence and absence of inhibitory compounds were subjected to sucrose density gradient analysis. Samples (150 μL) were laid on sucrose density gradients composed of 800 μL of 50% (wt per vol), 800 μL of 40%, 800 μL of 30%, 800 μL of 20%, and 650 μL of 10% sucrose in 150 mM HEPES, pH 7.5. After centrifugation at 250,000 × *g* and 20 °C for 1.5 h, ten 400 μL fractions were collected from top to bottom, and each fraction was analyzed by 15% SDS-PAGE. The gels were stained with Coomassie Brilliant Blue R-250 or subjected to immunoblot analysis with anti-HBV core antibody. The densities of the individual bands were analyzed by ImageJ software.

### Electron microscopy of HBV capsid assembly in vitro

Cp149 was assembled in reaction buffer (150 mM HEPES, pH 7.5, 15 mM NaCl) in the presence and absence of ciclopirox. Five microliters of the solution containing the assembled HBV core particles was negatively stained by incubation on a carbon-coated grid for 1 min, followed by washing with water and staining with 2% uranyl acetate for 1 min. The grids were examined with a Tecnai G2 F30 S-TWIN transmission electron microscope.

### Crystallization and data collection

To co-crystallize HBV core protein with ciclopirox, concentrated Cp149-Y132A protein (50 mg per mL) was incubated with 5 mM ciclopirox on ice for 30 min in a buffer consisting of 20 mM Tris HCl pH 9.0 and 200 mM NaCl. Crystals of HBV core protein and ciclopirox were grown by the sitting-drop vapor diffusion method at 22 °C with a reservoir solution containing 100 mM ammonium citrate pH 6.0–7.0, 2–14% (wt per vol) PEG5000MME, 2% hexylene glycol, and 10% isopropanol. For data collection, the protein crystals were flash-frozen in liquid nitrogen with 20% glycerol, added as a cryoprotectant. X-ray diffraction datasets were collected at beamline 7A of the Pohang Accelerator Laboratory (PAL). The diffraction data were indexed, integrated, and scaled with the HKL2000 program package (HKL Research Inc.).

### Structure determination

The initial phases were calculated by molecular replacement using the program Phaser^57^. The previously published structure of the HBV core protein complexed with NVR-010–001-E2 (PDB code 5E0I) was used as search probe^28^. Atomic models were built by iterative modeling and refinement using the programs COOT^58^, REFMAC5^59^, and PHENIX^60^. All molecular figures were prepared with PyMol (www.pymol.org). The crystallographic data and refinement statistics are summarized in Table 1.

### Cell viability

HepG2.2.15 cells and HepG2 cells transfected with the pHBV1.2× plasmid were seeded on 96-well plates at a density of 2 × 10^4^ cells per well. After treatment with the indicated compound(s), the medium was removed and the cells were incubated at 37 °C for 1 h in EZ-CYTOX solution (EZ-5000, DoGenBio). The absorbance at 450 nm was then measured.

### ELISAs

An HBsAg ELISA kit (KA0286, Abnova) was used to quantify HBsAg protein. Briefly, culture media were incubated in individual wells of the capture plates for 2 h. After washing, horseradish peroxidase-labeled rabbit anti-HBV surface antibody was added, followed by incubation for an additional 2 h. The substrate solution was added for 15–60 min and absorbance at 450 nm was measured.

### Mouse model

Human liver-chimeric uPA/SCID (PXB) mice were generated by PhoenixBio Co., Ltd. (Higashi-Hiroshima, Japan)^61^. At age 3 weeks, uPA/SCID mice underwent transplantation of human hepatocytes (10^5^–10^6^ cells per mouse) via the spleen. At 9 weeks after transplantation, the replacement index in all the mice exceeded 90%. At age 17 weeks, the mice were transferred to the laboratory animal research center of CHA University where they received a single intravenous injection of human HBV virions (5 × 10^7^ copies per mouse, genotype D). After 6 weeks, persistent HBV infection of the human hepatocytes was established in the chimeric liver. At 22 weeks of age the mice began daily oral treatment with ciclopirox (5 mg per kg). TDF (5 mg per kg) was also administered orally every day. Serum samples were collected from the orbital sinus each week to measure HBsAg (WB-1296, Wantai Bio-Pharm), HBeAg (WB-2496, Wantai Bio-Pharm), HBV DNA, ALT (ab234578, Abcam), and human albumin (ab179887, Abcam). The animals were killed by CO_2_ gassing after 35 days. Liver tissue samples were analyzed to measure cccDNA, and sectioned for immunohistochemical staining with anti-HBV core antibodies (OAAI00128, Aviva Systems Biology). Intrahepatic cccDNA levels were determined in DNA extracted from liver specimens using a G-spin™ Total DNA extraction kit (17046, Intron) after digestion with T5 nuclease (10 U per μg for 0.5 h). For normalization, numbers of human hepatocytes in the liver specimens were estimated by measuring human hemoglobin beta (assay ID Hs00758889_s1, Thermo Fisher Scientific, USA). Animal procedures were performed in accordance with the guidelines of the Committee for Animal Experiments of CHA University. Infection, extraction of serum samples, and sacrifice were performed under ether anesthesia. All animal protocols were approved by the Institutional Animal Care and Use Committee of CHA University (IACUC-180090).

### Statistical analysis

The bar-graph data show the means and standard deviations of one representative of 2–3 independent experiments. Groups were compared using two-tailed student’s *t*-test. *P* values of < 0.05 were considered significant.

### Reporting summary

Further information on research design is available in the Nature Research Reporting Summary linked to this article.

## Supplementary information

Supplementary information

Reporting Summary

Source Data

## Data Availability

The crystal structure presented in this work has been deposited in the Protein Data Bank (PDB) with accession code 6J10. A reporting summary for this article is available as a Supplementary Information file. The source data underlying Fig. 1–7 and Supplementary Figs. 1, 2, 8, 11–15 are provided as a Source Data file. Other data supporting the findings of this manuscript are available from the corresponding authors upon request.
