# Supplementary material for: Ciclopirox inhibits Hepatitis B Virus secretion by blocking capsid assembly
Source: Nat Commun. 2019 May 16;10:2184. doi: 10.1038/s41467-019-10200-5 (PMC6522524; doi:10.1038/s41467-019-10200-5)
Supplement: Supplementary file 1 — Supplementary information [file 41467_2019_10200_MOESM1_ESM.pdf]

Supplementary information

Ciclopirox inhibits Hepatitis B Virus secretion by blocking capsid assembly

Jung-Ah Kang,[1,*] Songwon Kim,[1,*] Minji Park,[2] Hyun-Jin Park,[1] Jeong-Hyun Kim,[1] Sanghyeok Park,[1] Jeong-Ryul Hwang,[1] Yong-Chul Kim,[1] Yoon Jun Kim,[3] Yuri Cho,[2] & Mi Sun Jin,[1] & Sung-Gyoo Park[1]

[1] School of Life Sciences, Gwangju Institute of Science and Technology, Gwangju 61005, Republic of Korea

[2] Department of Internal Medicine, CHA Gangnam Medical Center, CHA University School of Medicine, Seoul, 06125, Republic of Korea

[3] Department of Internal Medicine and Liver Research Institute, Seoul National University College of Medicine, Seoul, 03080, Republic of Korea

[*] These authors contributed equally to this work.

Correspondence and requests for materials should be addressed to Y.C. (email: yuricho@cha.ac.kr) or to M.S.J. (email: misunjin@gist.ac.kr) or to S.G.P. (email: sgpark@gist.ac.kr)

Supplementary Fig. 1. Effect of ciclopirox on pre-assembled HBV capsids.

a Time-dependent *in vitro* HBV capsid assembly measured by immunoblot analysis. **b** Pre-assembled HBV capsids were incubated with various concentrations of ciclopirox, and disruption of capsid assembly was measured by immunoblot analysis. The data in (a – b) are expressed as means ± SDs. The error bars represent the ± SD. Source data are provided as a Source Data file.

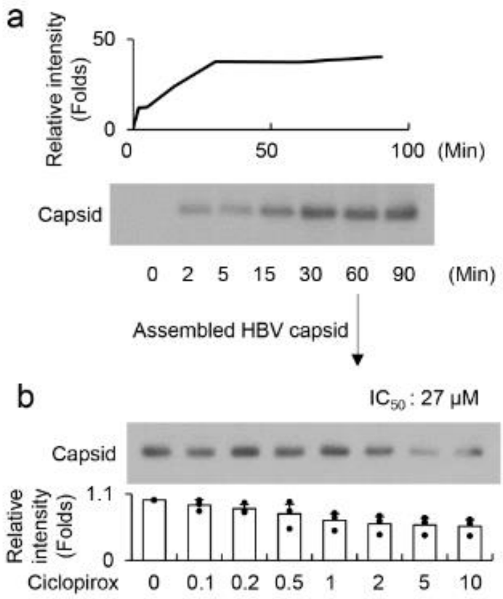

Supplementary Fig. 2. Effect of ciclopirox on intracellular HBV core protein stability.

Effect of ciclopirox on intracellular HBV capsid assembly and total HBV core protein. Huh-7 cells were transfected with pCDNA3-Core and exposed after 12 h to the indicated concentrations of ciclopirox for the indicated times. The cells were lysed and assembled HBV core particles were isolated by sucrose step-gradient ultracentrifugation, resolved on 1 % agarose gels, and subjected to immunoblot analysis with anti-HBV core antibody. Total intracellular HBV core protein was also determined by immunoblot analysis in the cell lysates. Core mRNA levels were measured by quantitative RT-PCR. The data expressed as means ± SDs. The error bars represent the ± SD. Source data are provided as a Source Data file.

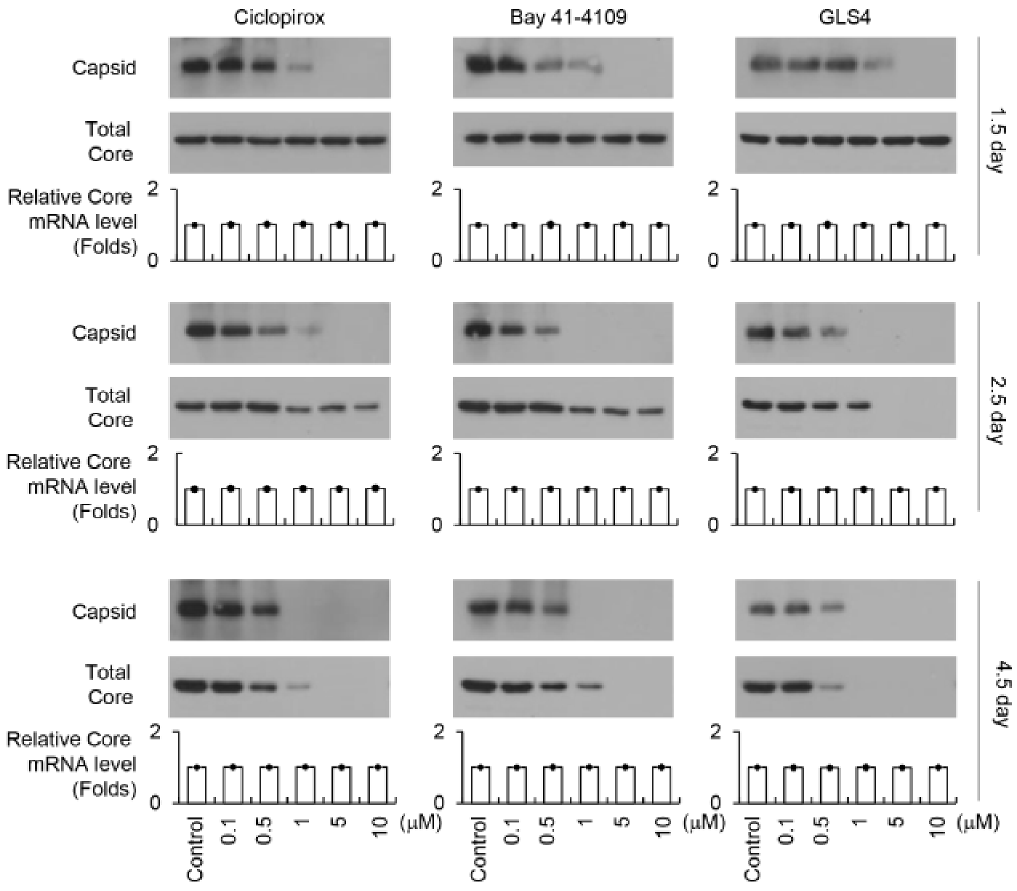

Supplementary Fig. 3. Superposition of the wild-type capsid structures (chains CDCDCD) and the Cp149-Y132A structures formed in the presence of ciclopirox.

a – b Hexamers (a) and dimers (b) of Cp149-Y132A formed in the presence of ciclopirox (PDB ID 6J10) were superimposed on those of native capsids (PDB ID 1QGT). The black triangle indicates the icosahedral threefold axis of the HBV capsid. Ciclopirox is shown as a space-filling model.

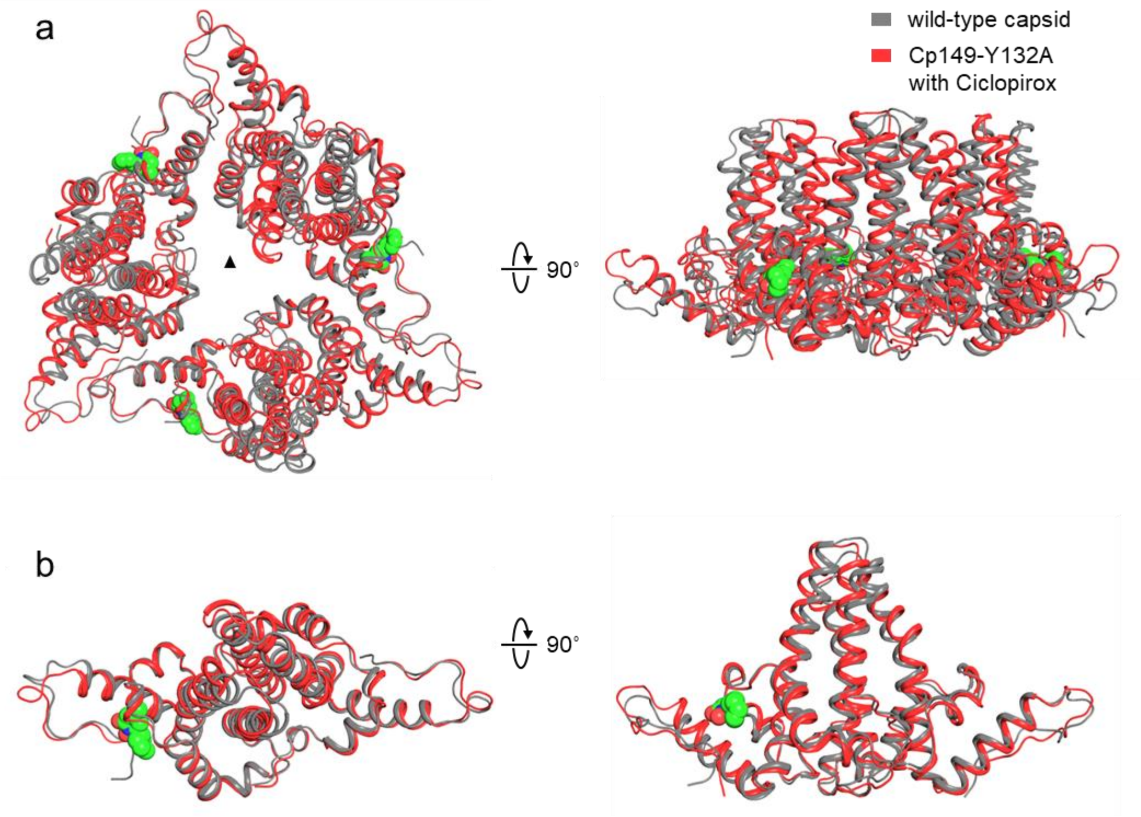

Supplementary Fig. 4. Crystal packing of ciclopirox-bound HBV core protein.

The unit cells are drawn as red boxes. The protomers of the hexameric HBV core protein in the asymmetric unit are colored light orange, orange, light blue, blue, pink, and red, respectively.

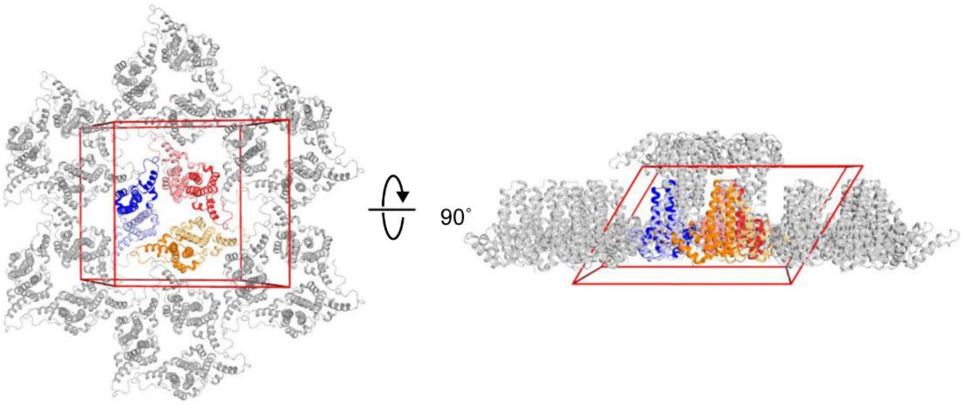

Supplementary Fig. 5. Structure alignment of protomers and dimers in the ciclopirox-bound HBV core protein hexamer.

a – **b** The structures of the three dimers (a) and six protomers (b) in the hexamer are superimposed. The color code is the same as in Supplementary Fig. 4. Notable structural differences are marked with black arrows.

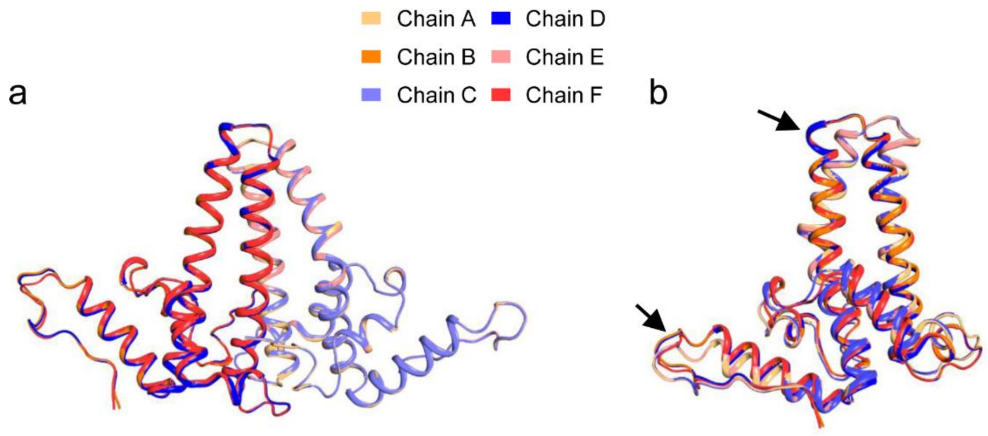

Supplementary Fig. 6. Superimposition of the dimer structures in the ciclopirox-bound HBV core protein on the previously-reported structures of HBV core protein.

a – **e** The dimers of (a) HBV core protein (apo, PDB code 4BMG) [1], complexed to (b) NVR-010–001-E2 (PDB code 5E0I) [2], (c) 4-Methyl HAP (PDB code 5GMZ) [3], (d) SBA_R01 (PDB code 5T2P) [4], and (e) HAP_R01 (PDB code 5WRE) [4] are superimposed on the ciclopirox-bound HBV core protein. The compounds are omitted for clarity.

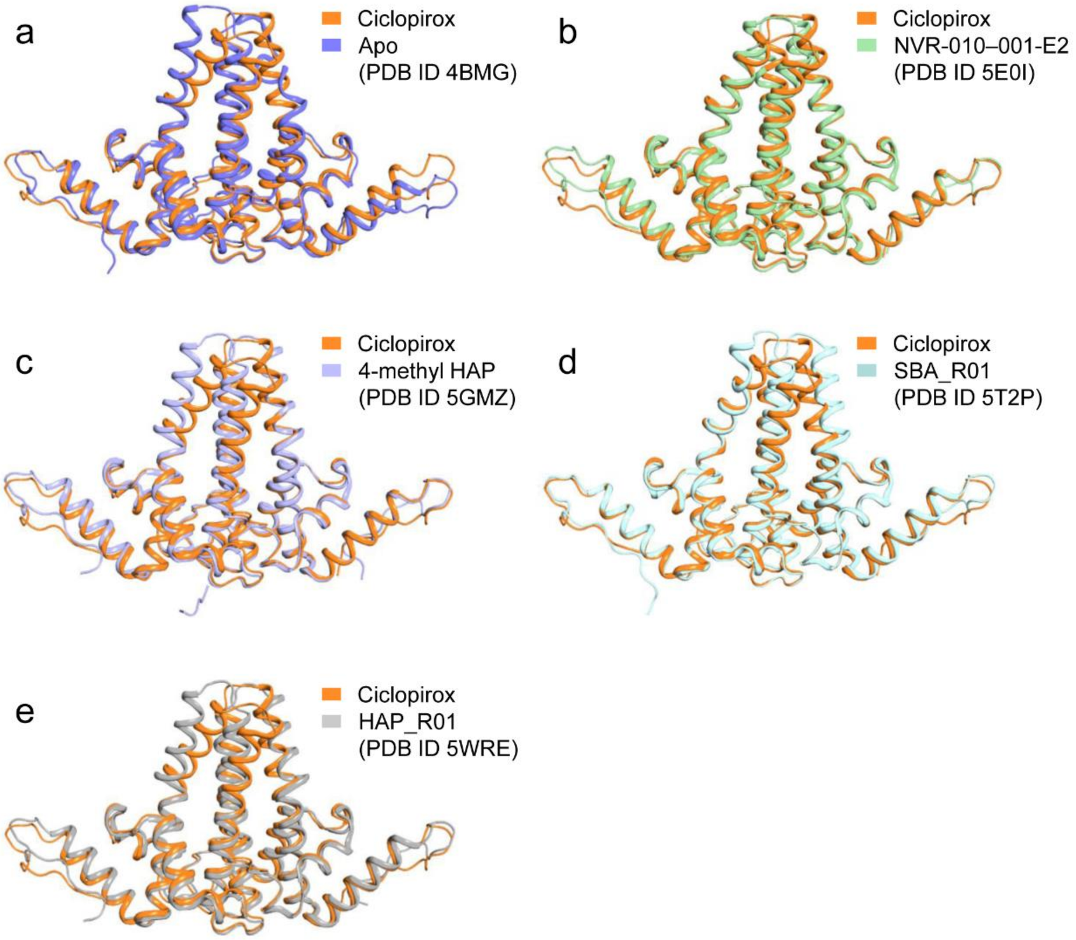

Supplementary Fig. 7. Stereo view of the ciclopirox binding site in the Cp149-Y132A structure.

Fo-Fc maps (contoured at 2.5 σ) of the ciclopirox binding site. The residues involved in ciclopirox binding are shown as orange stick models. Hydrogen bonds and salt bridges are indicated by dashed red lines.

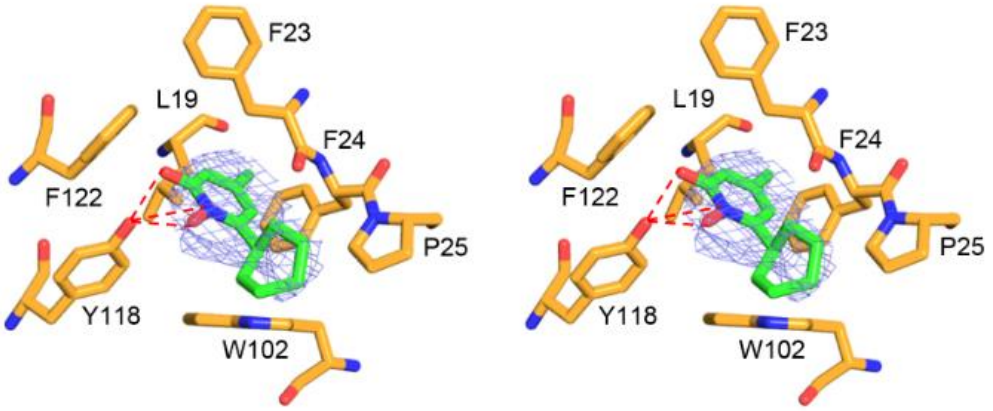

Supplementary Fig. 8. Effects of chemical modifications of ciclopirox on its inhibition of HBV capsid assembly.

Effects of ciclopirox and its chemical derivatives (6-cyclohexyl-4-methylpyridin-2(1H)-one, 6-cyclohexyl-4-methyl-2-pyrone, and (RS)-2-(3-cyclohexyl-5-methyl-4,5-dihydroisoxazol-5-yl)acetic Acid) on *in vitro* HBV capsid assembly. Various concentrations of either ciclopirox or its chemical derivatives were mixed with Cp149 in reaction buffer. After 1 h the mixtures were subjected to immunoblot analysis with anti-HBV core antibody. N.A., not available. The data are expressed as means ± SDs. The error bars represent the ± SD. *$p < 0.05$ by unpaired two-tailed Student's *t*-test. Source data are provided as a Source Data file.

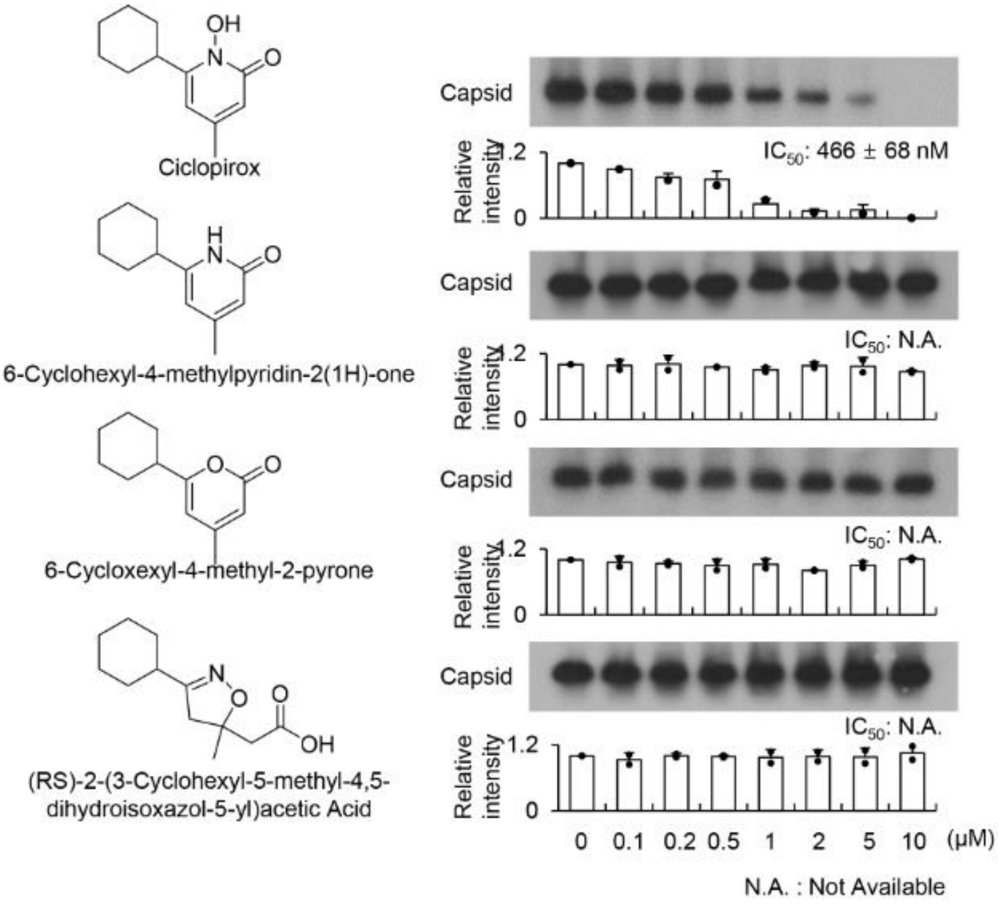

Supplementary Fig. 9. Comparisons of the binding modes of ciclopirox and other compounds.

a – **d** The hydrophobic pockets of HBV core proteins bound to (a) NVR-010–001-E2 (light green), (b) 4-Methyl HAP (light purple), (c) SBA_R01 (light cyan), and (d) HAP_R01 (gray) are shown and superimposed on the hydrophobic pockets of the ciclopirox-bound HBV core protein (orange). The compounds are shown as stick models. Ciclopirox and the other compounds are colored green and yellow, respectively. The rotations of F23s are marked by red arrows.

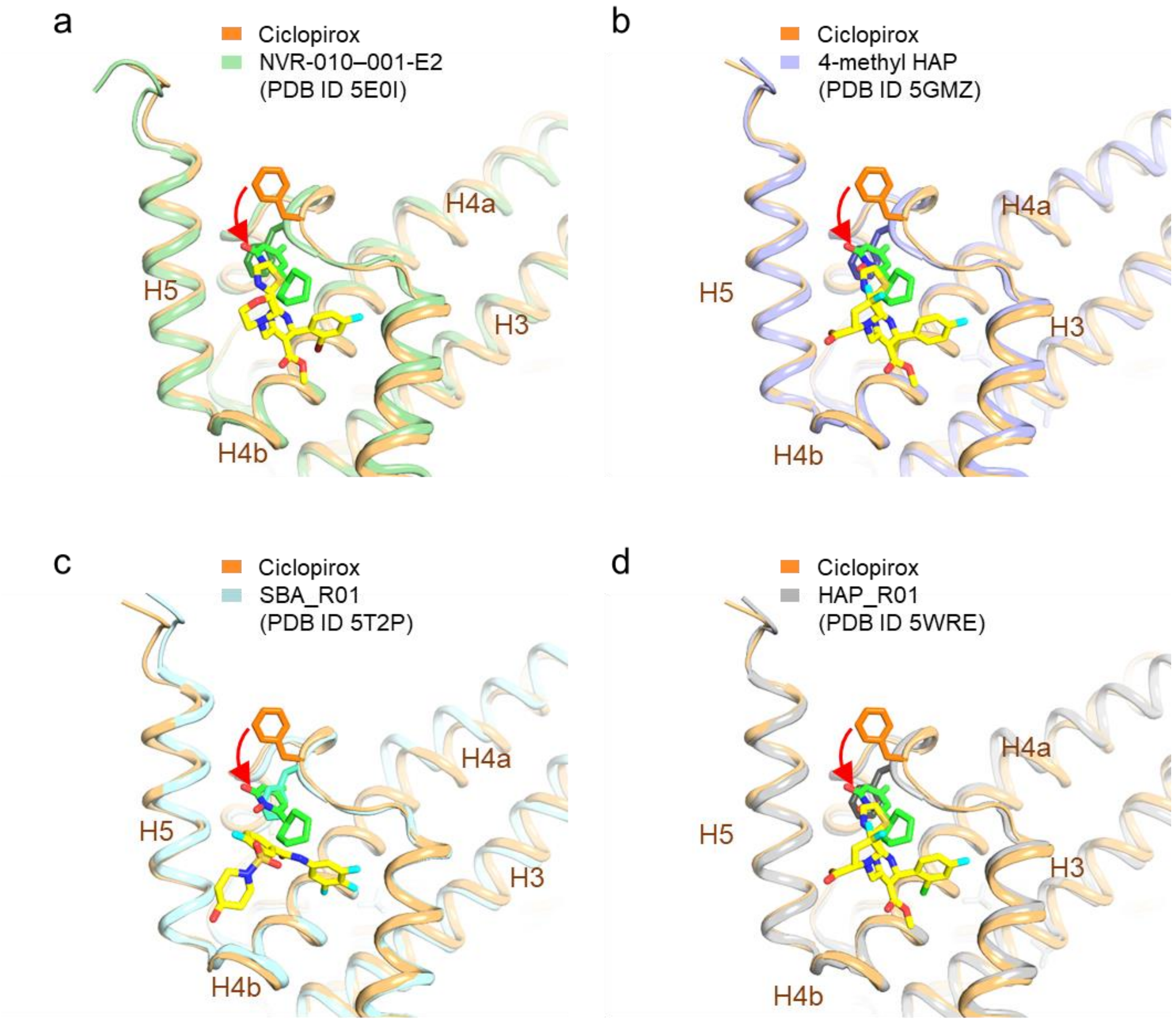

Supplementary Fig. 10. Comparisons of the proline-rich loop 6s in HBV core protein bound to ciclopirox and to other compounds.

a – d The hydrophobic pockets of HBV protein bound to (a) NVR-010–001-E2-bound (light green), (b) 4-Methyl HAP (light purple), (c) SBA_R01 (light cyan), and (d) HAP_R01 (gray) are shown and superimposed on the hydrophobic pocket of the ciclopirox-free protein (orange). The compounds are shown as stick models. Ciclopirox and the other compounds are colored green and yellow, respectively. The movements of proline-rich loop 6 are marked by red arrows.

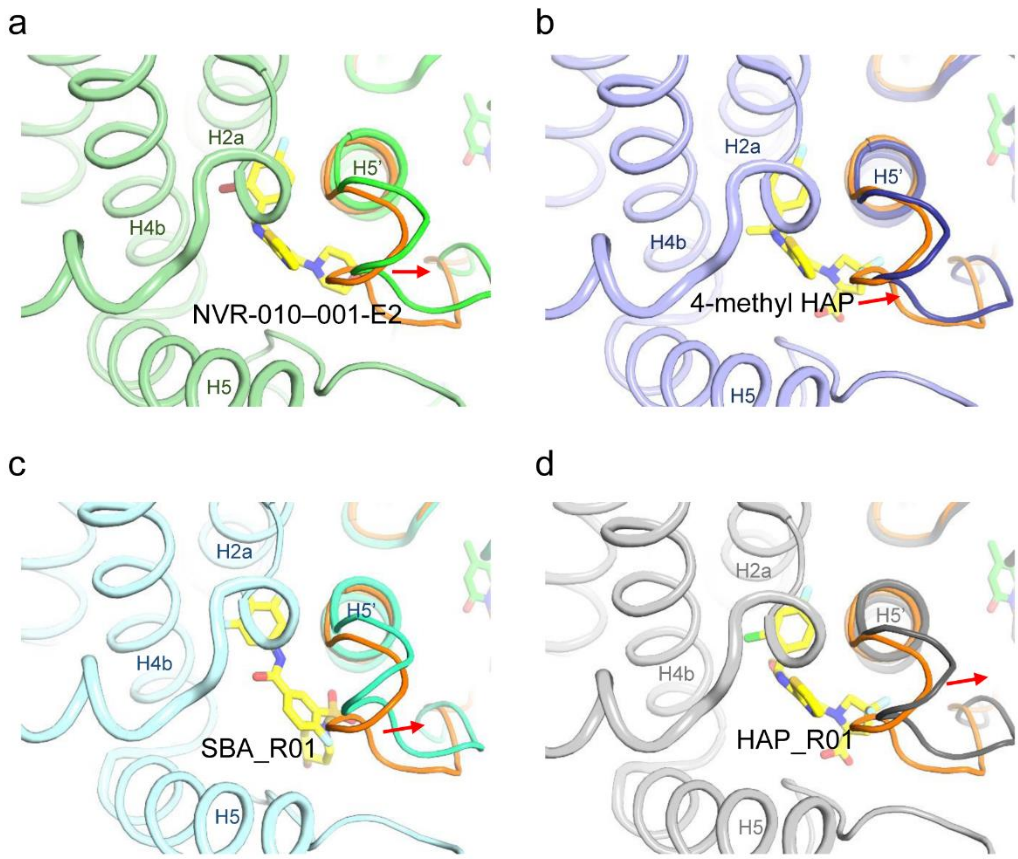

Supplementary Fig. 11. Effect of FBS serum iron level on Huh-7, HepG2, and HepG2.2.15 cell viability.

a Cells were maintained in medium supplemented with 2% FBS or 10% FBS for 1 day and treated with various concentrations of ciclopirox. After 6 days, cytotoxicity was measured. **b** Cells were treated with various concentrations of ciclopirox with or without 60 μM deferoxamine. After 6 days, cell cytotoxicity was measured. The data in (a – b) are expressed as means ± SDs. The error bars represent the ± SD. *$p < 0.05$ by unpaired two-tailed Student's *t*-test. Source data are provided as a Source Data file.

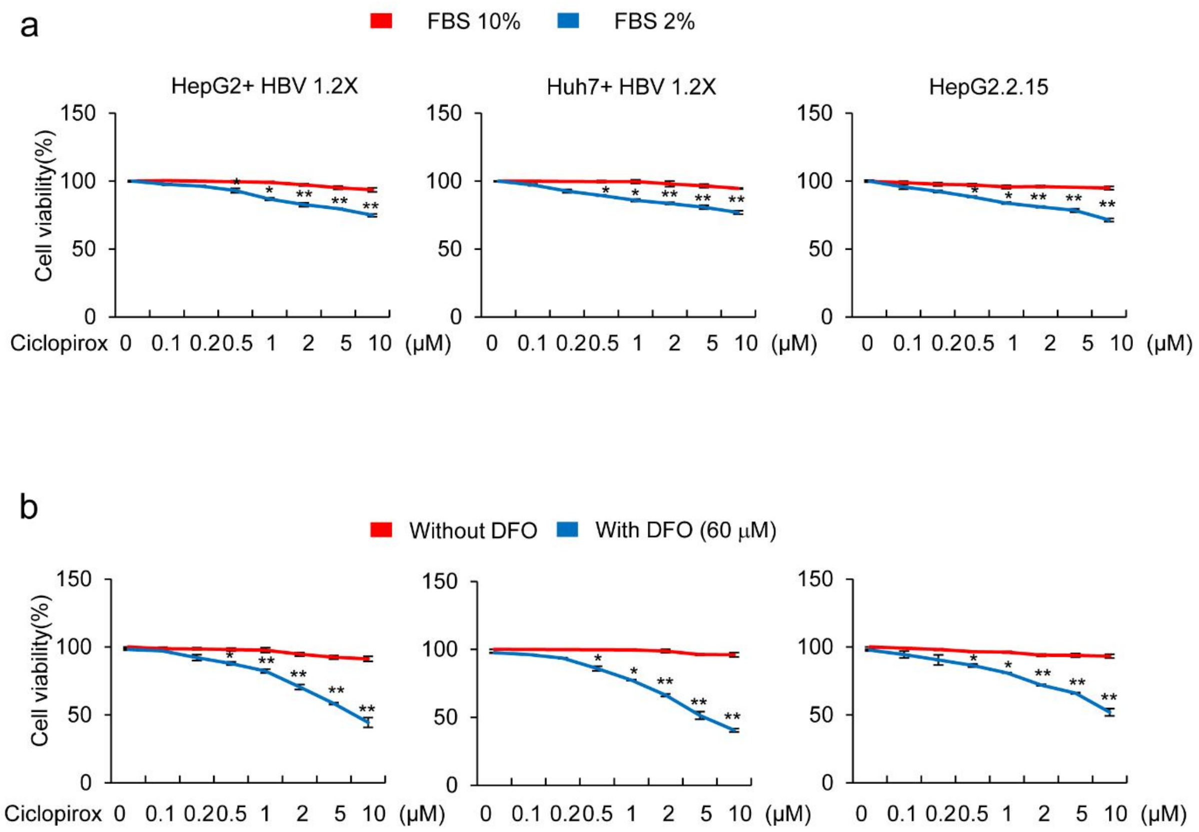

Supplementary Fig. 12. Effect of ciclopirox on HBV transcription.

a Schematic depiction of the R1, R2, and R3 regions of the HBV genome that were targeted by quantitative RT-PCR to quantitate the transcription of pgRNA, two HBV surface RNAs, and HBx RNA. RNA was extracted 6 days after treatment with various concentration (0.1 – 10 μM) of ciclopirox. **b** – **c** Region 3 transcription in ciclopirox-treated (b) HepG2.2.15 cells and (c) HepG2 cells transfected with the pHBV1.2× plasmid. **d** The promoter analysis of ciclopirox-treated HepG2 cells transfected with pGL3/HBV pgRNA promoter or pGL3/HBV sRNA promoter. The data shown in (b – d) represent two independent experiments and are expressed as means ± SDs. The error bars represent the ± SD. Source data are provided as a Source Data file.

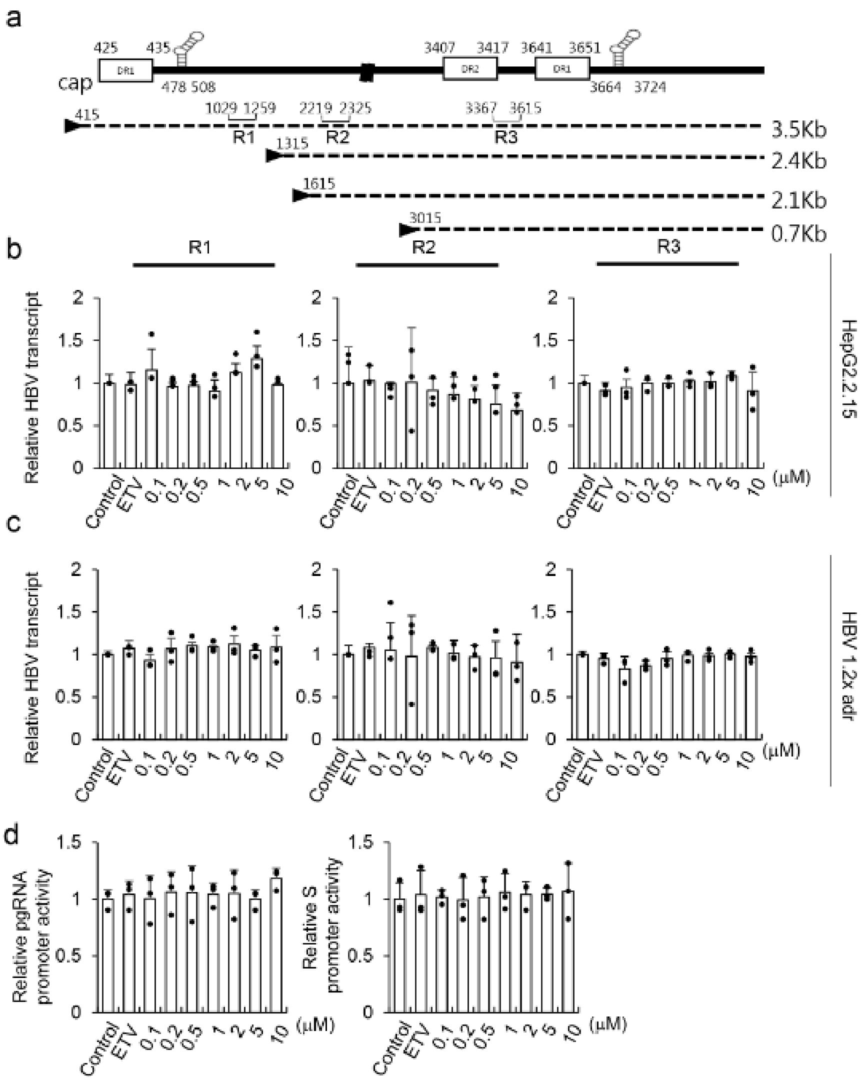

Supplementary Fig. 13. Effects of ciclopirox and TDF on cccDNA levels in HBV-infected cells. HepG2-NTCP or Huh-7-NTCP cells were infected with HBV and treated with either ciclopirox, TDF, or both. Quantitative PCR with an HBV DNA primer and probe set was used to measure intracellular cccDNA. The data are expressed as means ± SDs. The error bars represent the ± SD. Source data are provided as a Source Data file.

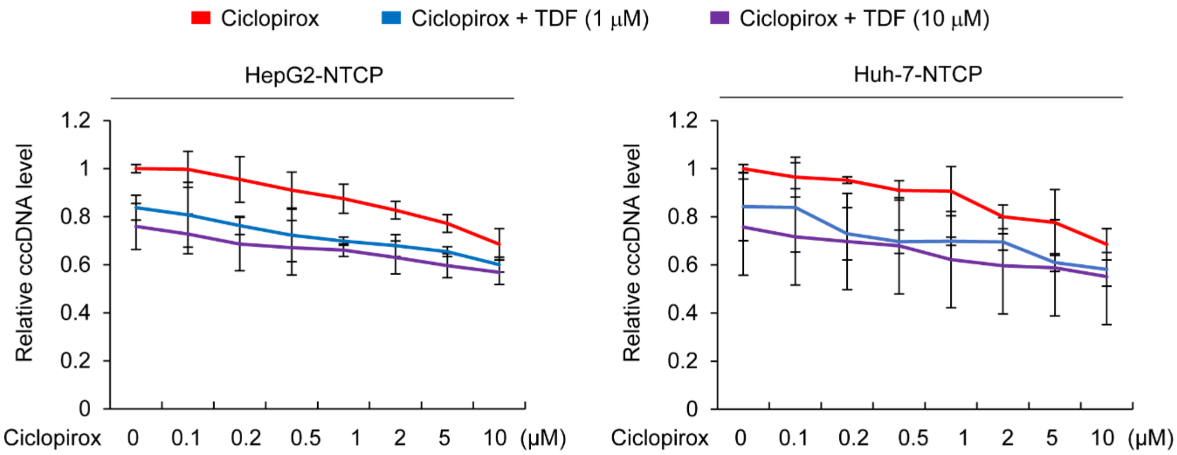

Supplementary Fig 14. T5 nuclease digestion facilitates specific detection of cccDNA

a Intracellular cccDNA was extracted from HBV-infected HepG2-NTCP or -Huh7-NTCP cells by the Hirt method, and digested with T5 nuclease (10 U per μg for 0.5 h) or left undigested. **b** Intracellular HBV DNA extracted from HepG2.2.15 cells and intracellular cccDNA extracted from HBV-infected HepG2-NTCP or -Huh7-NTCP cells was treated with T5 nuclease (10 U per μg for 0.5 h). *Eco*RI was used to linearize the cccDNA. The DNA samples (a-b) were then resolved on an agarose gel, and the HBV DNAs were detected by southern blotting using biotin-labeled HBV fragments. Source data are provided as a Source Data file.

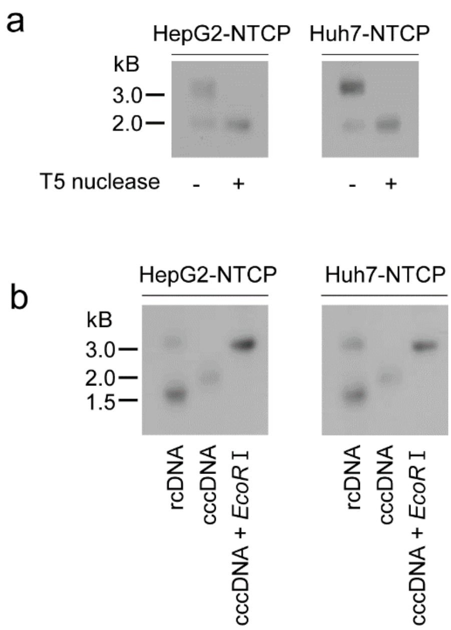

Supplementary Fig. 15. Antiviral activity of ciclopirox in a hydrodynamic injection mouse model. **a** Schematic depiction of the experiment. Six-week-old male C57BL/6 mice (n = 5 per group) were hydrodynamically injected via the tail vein with 25 μg of the HBV replicative plasmid pAAV-HBV 1.2× in a volume of PBS equivalent to 10 % of mouse body weight. The total volume of HBV DNA was delivered into the portal vein at high pressure within 4 – 6 seconds. Starting the day after plasmid injection, the mice were daily injected orally with TDF and/or ciclopirox (both 5 mg per kg, daily), and serum samples were taken from the orbital sinus. **b** Immunohistochemical analysis of hepatitis B core antigen (HBcAg) expression in liver sections obtained 5 days after plasmid injection (200× magnification). **c** Serum HBV genomic DNA levels measured by quantitative PCR. **d** Serum HBsAg levels determined by ELISA. Protocols were approved by the Institutional Animal Care and Use Committee of CHA University (IACUC-170049). The data in (c – d) are expressed as means ± SDs. The error bars represent the ± SD. Scale bars, 100 μm. $^*p < 0.05$ by unpaired two-tailed Student's *t*-test. Source data are provided as a Source Data file.

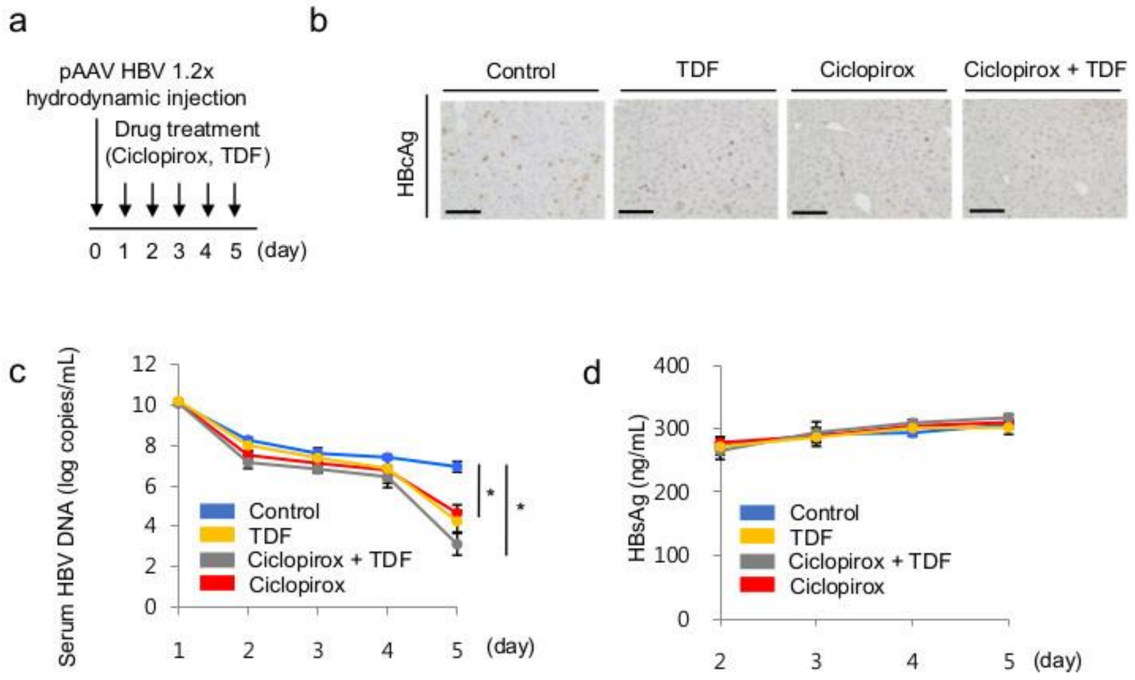

Supplementary Fig. 16. Model of how ciclopirox induces abnormal HBV capsid assembly.

In the absence of ciclopirox, HBV core protein forms dimers, and these assemble into HBV capsids. In the presence of ciclopirox, normal HBV capsid assembly is blocked. In addition, ciclopirox can induce disassembly of assembled capsids.

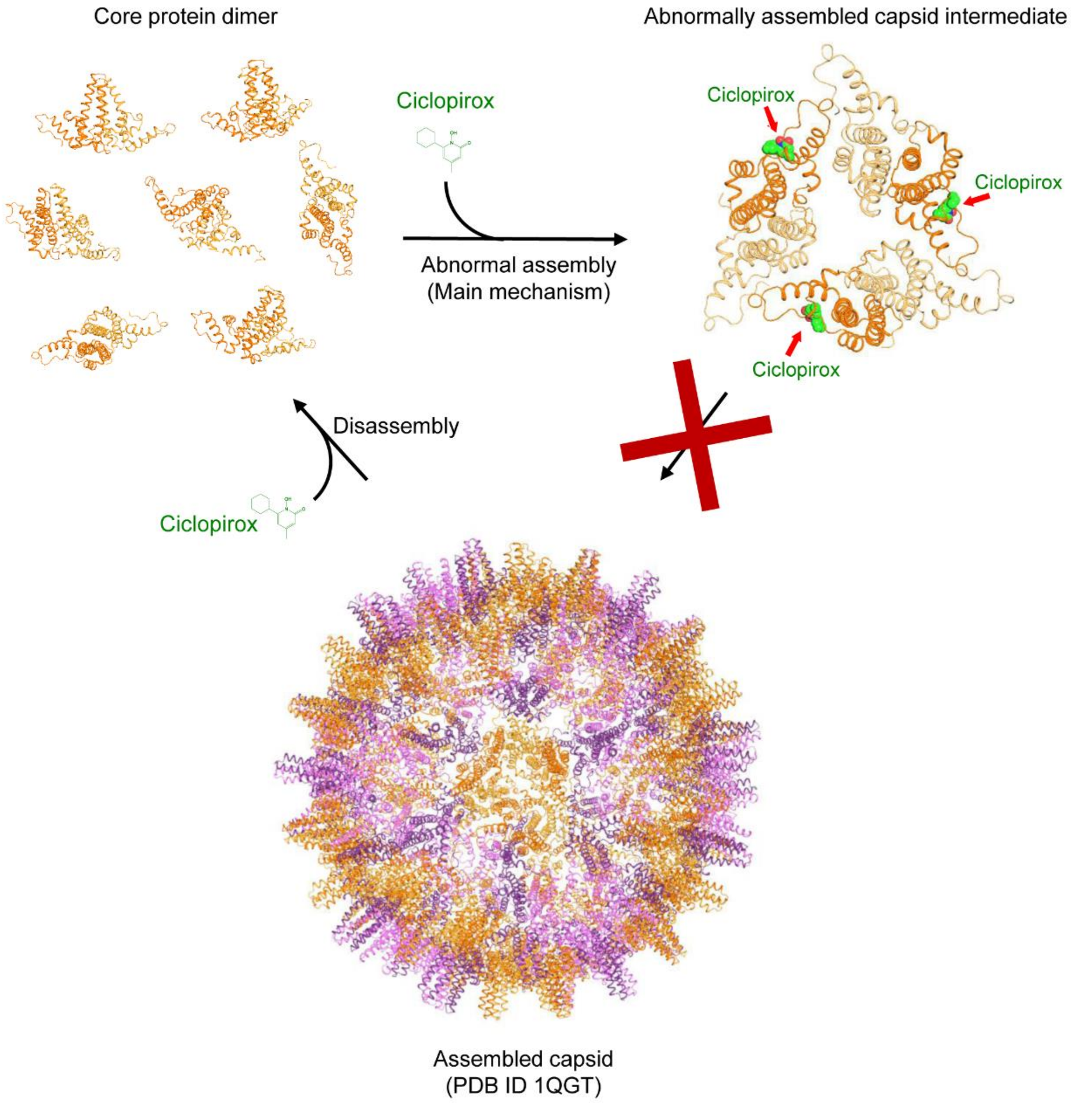

Supplementary Table 1. List of primers used in this study

Name	Sequence
HBV DNA primer and probe set (R2)	TCC TCT TCA TCC TGC TGC TAT G
HBV DNA primer and probe set (R2)	CGT GCT GGT AGT TGA TGT TCC T
HBV DNA primer and probe set	TAT TGG TTC TTC TGG ACT A
cccDNA primer and probe set	TTC TCA TCT GCC GGA CCG
cccDNA primer and probe set	CAC AGC TTG GAG GCT TGA AC
cccDNA primer and probe set	CCT AAT CAT CTC TTG TTC AT
HBx-encoding RNA (R3)	CCG TCT GTG CCT TCT CAT CTG C
HBx-encoding RNA (R3)	ACC AAT TTA TGC CTA CAG CCT CC
HBV core	TTC GCA CTC CTC CCG CTT AC
HBV core	GAG GCG AGG GAG TTC TTC TT
Human GAPDH	GGA GCG AGA TCC CTC CAA AAT
Human GAPDH	GGC TGT TGT CAT ACT TCT CAT G
pgRNA and precore RNA (R1)	ATA TCT CGA GGG GAG GAG ACT AGG TTA AAG
pgRNA and precore RNA (R1)	GCA GAT CTG AAC ATG AGA TGA TTA GGC A

Supplementary Table 2. The 19 FDA-approved compounds that reduced HBV DNA secretion by HepG2.2.15 cells.

Number	Drug name	Indication	Compounds selected by initial screening (3days)	% of inhibition (ETV =100%)	Confirmation of initially screened compounds (6days)	% of inhibition (ETV =100%)	Reduction of pgRNA level	Inhibition of HBV capsid assembly	https://www.drugbank.ca/		Ref
									pKa value	Human oral administration	
S#1	Ftorafur	Cancer	o	130.8	o	64.7			8.08(acidic), -4.3(basic)	Capsule	5
S#2	Acitretin	Metabolic disease	o	109.9		1.8			5.01(acidic), -4.8(basic)	Capsule	-
S#3	Nebivolol (Bystolic)	Cardiovascular disease	o	102.9	o	78.4			13.52(acidic), 8.9(basic)	Tablet	6
S#4	Suplatast tosylate	Cardiovascular disease	o	125.4	o	76.2			12.7(acidic)	–	7
S#5	Sotalol (Betapace)	Neurological disease	o	128.5	o	69.7			10.07(acidic), 9.43(basic)	Tablet	8
S#6	Tenoxicam (Mobiflex)	Inflammation	o	110.4	o	79.4	o		2.21(acidic), 4.26(basic)	Tablet	9
S#7	Ciclopirox (Penlac)	Infection	o	142.5	o	88.6		o	6.84(acidic), -6.2(basic)	Solution	10
S#8	Phenytoin sodium (Dilantin)	Neurological disease	o	124.4		-2.4			9.47(acidic), -9(basic)	Capsule, Tablet, Suspension	-
S#9	L-Adrenaline (Epinephrine)	Cardiovascular disease	o	152.6		1.5			9.69(acidic), 8.91(basic)	X, Injection	-

Number	Drug name	Indication	Compounds selected by initial screening (3days)	% of inhibition (ETV =100%)	Confirmation of initially screened compounds (6days)	% of inhibition (ETV =100%)	Reduction of pgRNA level	Inhibition of HBV capsid assembly	https://www.drugbank.ca/		Ref
									pKa value	Human oral administration	
S#10	Dopamine hydrochloride (Inotropin)	Infection	o	162.3	o	67.9			10.01(acidic), 9.27(basic)	X, Injection	11
S#11	Isoconazole nitrate (Travogen)	Infection	o	147.7	o	41.4			6.77(basic)	–	12
S#12	Econazole nitrate (Spectazole)	Neurological disease	o	147	o	27.5	o		6.77(basic)	X, Cream, Aerosol	13
S#13	Miconazole (Monistat)	Neurological disease	o	131.8		-2.8			6.77(basic)	Tablet	-
S#14	Tiotropium Bromide hydrate	Neurological disease	o	116.9		4.9			10.35(acidic), -4.3(basic)	Capsule	-
S#15	Amidopyrine	Inflammation	o	111.6		7.1			3.47(basic)	–	-
S#16	Sodium nitrite	Neurological disease	o	152.7	o	83.1	o		3.32(acidic), -3.5(basic)	X, Injection	14
S#17	Rotigotine	Neurological disease	o	104.5	o	65.9			10.03(acidic), 10.97(basic)	X, Patch	15
S#18	Procyclidine HCl	Neurological disease	o	111.7	o	70.4			13.84(acidic), 9.45(basic)	Tablet	16
S#19	Terfenadine	Neurological Disease	o	100.7	o	83.6	o		13.2(acidic), 9.02(basic)	Tablet	17

Supplementary Table 3. Mean numbers of blood cells, and parameters for kidney and liver toxicity induced by treatment with oral ciclopirox at concentrations of 0, 1, 5 mg per kg in daily for 4 weeks.

	Ciclopirox dose		
	0 mg/kg/day	1 mg/kg/day	5 mg/kg/day
WBC (× 10^3/μL)	5.6 ± 1.4	4.4 ± 1.0*	5.9 ± 2.1*
RBC (× 10^6/μL)	10.5 ± 0.2	9.6 ± 0.9*	10.6 ± 0.1*
Hemoglobin (g/dL)	16.1 ± 0.5	16.1 ± 1.2*	16.9 ± 0.4*
Platelets (× 10^3/μL)	1451.3 ± 30.0	1098.3 ± 108.3*	1221.7 ± 156.1*
AST (IU/L)	144 ± 10.6	146.3 ± 7.8*	135.0 ± 17.3*
ALT (IU/L)	86.7 ± 11.5	88.3 ± 15.3*	83.3 ± 5.8*
ALP (IU/L)	52.3 ± 8.7	46.0 ±5.3*	42.0 ± 6.2*
Albumin (g/dL)	3.7 ± 0.3	4.3 ± 0.3*	3.7 ± 0.3*
Total protein (g/dL)	6.2 ± 0.3	7.3 ± 0.3*	6.5 ± 0.5*
Total cholesterol (mg/dL)	61.7 ± 7.6	73.3 ± 7.6*	58.3 ± 7.6*
Triglyceride (mg/dL)	185.7 ± 1.5	194.3 ± 4.0*	182.0 ± 3.6*
Glucose (mg/dL)	155.3 ± 5.0	167.7 ± 10.8*	155.3 ± 6.8*
Creatinine (mg/dL)	1.0 ± 0.0	0.8 ± 0.3*	0.7 ± 0.6*
BUN (mg/dL)	20.0 ± 0.0	21.7 ± 2.9*	20.0 ± 5.0*
Total bilirubin (mg/dL)	0.5 ± 0.0	0.8 ± 0.3*	0.8 ± 0.3*
Phosphorous (mg/dL)	11.5 ± 0.5	11.3 ± 1.6*	12.2 ± 3.4*
Calcium (mg/dL)	2.7 ± 0.8	1.2 ± 1.0*	2.0 ± 1.8*
Na (mmol/L)	144.3 ± 4.2	145.3 ± 0.6*	143.7 ± 1.2*
K (mmol/L)	4.9 ± 0.2	4.9 ± 0.1*	4.9 ± 0.1*
Cl (mmol/L)	99.3 ± 2.3	100.0 ± 1.0*	99.0 ± 1.0*
Urinalysis			
Urobilinogen	(–)	(–)	(–)
Glucose	(–)	(–)	(–)
Bilirubin	(–)	(–)	(–)
Ketones	(–)	(–)	(–)
S.G	1.06 ± 0	1.06 ± 0*	1.06 ± 0*
Blood	(–)	(–)	(–)
pH	6.5 ± 0	6.5 ± 0*	6.5 ± 0*
Protein	(–)	(–)	(–)
Nitrate	(–)	(–)	(–)
Leukocytes	(–)	(–)	(–)

Data are expressed as means ± standard deviations.
Abbreviations: WBC, white blood cell, AST, aspartate transaminase; ALT, alanine transaminase; ALP, alkaline phosphatase; BUN, blood urea nitrogen; Na, sodium; K, potassium; Cl. Chloride; S.G, specific gravity.*Not significant ($p > 0.05$) compared to the 0 mg/kg/day ciclopirox group

Supplementary references

1. Alexander CG, Jurgens MC, Shepherd DA, Freund SM, Ashcroft AE, Ferguson N. Thermodynamic origins of protein folding, allostery, and capsid formation in the human hepatitis B virus core protein. *Proceedings of the National Academy of Sciences of the United States of America* **110**, E2782-2791 (2013).

2. Klumpp K, *et al.* High-resolution crystal structure of a hepatitis B virus replication inhibitor bound to the viral core protein. *Proceedings of the National Academy of Sciences of the United States of America* **112**, 15196-15201 (2015).

3. Qiu Z, *et al.* Design and Synthesis of Orally Bioavailable 4-Methyl Heteroaryldihydropyrimidine Based Hepatitis B Virus (HBV) Capsid Inhibitors. *Journal of medicinal chemistry* **59**, 7651-7666 (2016).

4. Zhou Z, *et al.* Heteroaryldihydropyrimidine (HAP) and Sulfamoylbenzamide (SBA) Inhibit Hepatitis B Virus Replication by Different Molecular Mechanisms. *Sci Rep* **7**, 42374 (2017).

5. Hortobagyi GN, *et al.* Ftorafur, adriamycin, cyclophosphamide and BCG in the treatment of metastatic breast cancer. *Cancer* **44**, 398-405 (1979).

6. Hilas O, Ezzo D. Nebivolol (bystolic), a novel Beta blocker for hypertension. *P T* **34**, 188-192 (2009).

7. Shahriar M, *et al.* Suplatast tosilate inhibits histamine signaling by direct and indirect down-regulation of histamine H1 receptor gene expression through suppression of histidine decarboxylase and IL-4 gene transcriptions. *J Immunol* **183**, 2133-2141 (2009).

8. Dunnington CS. Sotalol hydrochloride (Betapace): a new antiarrhythmic drug. *Am J Crit Care* **2**, 397-406 (1993).

9. Tanaka Y, Maeda M, Nakamura K. [Pharmacologic studies on Ro 12-0068, a new non-steroidal

anti-inflammatory drug (author's transl)]. *Nihon Yakurigaku Zasshi* **77**, 531-552 (1981).

10. Sehgal VN. Ciclopirox: a new topical pyrodonium antimycotic agent. A double-blind study in superficial dermatomycoses. *Br J Dermatol* **95**, 83-88 (1976).

11. Gardella LA, Kesler H, Amann A, Carter JE. Intropin (dopamine hydrochloride) intravenous admixture compatibility, Part 3: stability with miscellaneous additives. *Am J Hosp Pharm* **35**, 581-584 (1978).

12. Oyeka CA, Gugnani HC. Isoconazole nitrate versus clotrimazole in foot and nail infections due to Hendersonula toruloidea, Scytalidium hyalinum and dermatophytes. *Mycoses* **35**, 357-361 (1992).

13. Daily AD, Kramer KJ, Rex IH, Thorne EG. Econazole nitrate (Spectazole) cream, 1 percent: a topical agent for the treatment of tinea pedis. *Cutis* **35**, 278-280 (1985).

14. Zeman C, *et al.* New questions and insights into nitrate/nitrite and human health effects: a retrospective cohort study of private well users' immunological and wellness status. *J Environ Health* **74**, 8-18 (2011).

15. Bunten S, Happe S. Rotigotine transdermal system: a short review. *Neuropsychiatr Dis Treat* **2**, 421-426 (2006).

16. Jevtovic-Todorovic V, Meyenburg AP, Olney JW, Wozniak DF. Anti-parkinsonian agents procyclidine and ethopropazine alleviate thermal hyperalgesia in neuropathic rats. *Neuropharmacology* **44**, 739-748 (2003).

17. Akagi M, Mio M, Miyoshi K, Tasaka K. Antiallergic effects of terfenadine on immediate type hypersensitivity reactions. *Immunopharmacol Immunotoxicol* **9**, 257-279 (1987).
